# Understanding the role of visceral fat in metabolically healthy versus unhealthy obesity: a sex-based analysis of the transcriptome

**DOI:** 10.1186/s13293-025-00777-6

**Published:** 2025-11-06

**Authors:** María Calderón-Domínguez, Isabel Sánchez-Muñoz, Raquel González-Blázquez, Marta Gil-Ortega, Beatriz Somoza, Ricardo Arroyo-Solera, Paloma Fernández, Esther Carrera, Javier Valverde-Pozo, María Larriva, Jose Miguel Cárdenas-Rebollo, Juan Carlos Ruiz de Adana, Marta Viana, Martín Alcalá

**Affiliations:** 1 Biomedicine, Biotechnology and Public Health Department, University of Cadiz, 11002 Cadiz, Spain; 2 Biomedical Research and Innovation Institute of Cadiz (INiBICA) Research Unit, Puerta del Mar University Hospital, 11009 Cadiz, Spain; 3 Research Department, Biomedical Research Foundation at Getafe University Hospital, 28905 Getafe, Spain; 4 Faculty of Medicine, Health and Sports. Department of Medicine, Universidad Europea de Madrid, Madrid, Spain; 5 Departamento de Ciencias Farmacéuticas y de La Salud, Facultad de Farmacia, Universidad San Pablo-CEU, CEU Universities., Urbanización Montepríncipe, 28660 Boadilla del Monte, Spain; 6 Instituto de Medicina Molecular Aplicada Nemesio Díez, Universidad San Pablo-CEU, CEU Universities, Madrid, Spain; 7 Departamento de Química y Bioquímica, Facultad de Farmacia, Universidad San Pablo-CEU, CEU Universities, Urbanización Montepríncipe, 28660 Boadilla del Monte, Spain; 8 Departamento de Matemáticas y Ciencias de Datos, Facultad de Ciencias Económicas y Empresariales, Universidad San Pablo-CEU, CEU Universities, Calle Julián Romea 23, 28003 Madrid, Spain

**Keywords:** Adipose tissue, Metabolically healthy obesity, Transcriptome, Inflammation, Extracellular matrix

## Abstract

**Background:**

The term “metabolically healthy obesity” is used to define those patients with obesity that do not present elements of metabolic syndrome. The causes behind this temporary reduction of the cardiovascular risk are still unknown, although these patients are characterized by a conserved expansion capacity of the adipose tissue, preventing ectopic accumulation of fat. Since hormones are key regulators in adipogenesis, we hypothesize that there are sex-specific differences in visceral white adipose tissue (vWAT) biology that may contribute to metabolic health disparities between men and women.

**Methods:**

60 patients attending the Morbid Obesity Unit from the Hospital Universitario de Getafe for elective bariatric surgery were enrolled. Prior to the surgery, a full biochemical panel was carried out. During the procedure, a portion of vWAT was excised and snap-frozen for histological analysis and for the study of the transcriptomic fingerprint in 8 metabolically healthy (MH) and 8 metabolically unhealthy (MU) patients using a transcript expression microarray. The results were validated by qPCR.

**Results:**

Functional enrichment analysis of the differentially expressed transcripts (DETs) revealed a similar vWAT transcriptome between MH and MU patients, with differences related to immune response and metabolism homeostasis. However, when we stratified the patients by sex, the number of DETs multiplied by 10, showing sex-specific signatures. MH men presented a reduced pro-inflammatory and oxidative stress profile in comparison to MU men. Thus, the transition from MH to a MU state in men led to a disruption of the normal biology of the tissue, which correlates to the apparition of comorbidities. Surprisingly, MH females exhibited the most deleterious profile, with alterations of key pathways related to inflammation, extracellular matrix organization and metabolism in comparison to MU females. Even those common processes (extracellular remodeling and inflammation) that were observed in men and women cohorts presented a unique signature. These results suggest that vWAT in females suffers an exaggerated pathological state in response to the increased demand to store energy in comparison to men.

**Conclusion:**

These findings suggest that obesity should be treated as a different entity in men and women and highlight the need of early intervention in female patients with obesity, even in the absence of comorbidities.

**Plain english summary:**

Obesity is often linked to metabolic problems, but some patients with obesity do not show typical signs of metabolic syndrome, a situation referred to as "metabolically healthy obesity." The reasons behind this are not fully understood, but it is thought that these individuals have healthier adipose tissue that prevents the accumulation of fat in other organs. Since hormones play an important role in fat storage, we examined if there are gender differences in how adipose tissue responds to fat accumulation. In this study, we analyzed the genes that were expressed in the visceral adipose tissue of patients with obesity who were metabolically healthy (MH) and those who were metabolically unhealthy (MU). When we compared the results, we found that men and women had different gene activity patterns. For men, MH adipose tissue had less inflammation and stress compared to MU adipose tissue. However, MH women tissue showed worse signs of inflammation, fibrosis and metabolism problems than MU women. Between genders, there are several differences in the pathways that are triggered by obesity, with women having a more pathological profile. Even those processes that were common had a worst profile than in men. This suggests that women adipose tissue does not adapt equally to the increased demand for energy storage, even in the absence of metabolic abnormalities. These results suggest that obesity should be treated differently in men and women and emphasize the importance of early intervention for women, even if they do not show metabolic alterations.

**Supplementary Information:**

The online version contains supplementary material available at 10.1186/s13293-025-00777-6.

## Highlights

Sex-Specific Differences: The study reveals significant sex-specific differences in visceral white adipose tissue (vWAT) biology, independently of the metabolic status.

Male vWAT Biology: MH men showed reduced pro-inflammatory and oxidative stress profiles compared to MU men, which correlates to fewer comorbidities.

Female vWAT biology: MH women exhibited the most harmful profile, with alterations in inflammation, metabolism, and extracellular matrix organization, indicating a pathological vWAT compared to both MU women and MH men.

Implications for Treatment: The findings suggest that obesity should be treated differently in men and women, emphasizing early intervention for women, even in the absence of comorbidities, due to their unique adipose tissue response.

**Supplementary Information:**

The online version contains supplementary material available at 10.1186/s13293-025-00777-6.

## Introduction

According to the World Health Organization, the prevalence of global obesity has shown a constant increase since 1975. In 2022, more than 890 million adults were obese, representing approximately 13% (11% of men and 15% of women) of the worlds population [1]. Regarding children and adolescents, cases suffered an 11-fold increase from 1975 to 2016, rising from 11 to 124 million [2]. The relevance of these numbers arises from the observations that estimate that up to 80% of obese adolescents will become obese adults [3]. Besides the psychosocial and economic implications of this epidemic, the health consequences of obesity in the young population are highly relevant [4]. On the one hand, young obese patients will experience a long-lasting exposition to the physiopathological factors that accompany obesity (chronic inflammation, fibrosis, oxidative stress, metabolic disbalance, among others), which may aggravate the symptoms of the associated diseases. On the other hand, the apparition of obesity-related conditions that were typically observed in adults is now becoming more prevalent in underage patients.

Obesity increases the risk of suffering cardiovascular diseases (hypertension, myocardial infarction, stroke, atherosclerosis), metabolic diseases (diabetes, dyslipidemia, fatty liver disease), skeletal and respiratory conditions, gestational and perinatal complications, and even some types of cancer. Although this statement might apply to the vast majority of obese patients, in some cases, body mass index is not related to poor health. Several studies have postulated the existence of a small proportion of patients who do not exhibit the typical comorbidities of obesity [5–8]. They are known as metabolically healthy obese (MH) and were first described in the 1950s, in contrast to those who present some of the former pathologies known as metabolically unhealthy obese (MU).

A recent population-based prospective cohort study of 381,363 patients revealed that a third of the MH patients who remained obese during the follow-up study turned into MU within 3 to 5 years [9]**.** This supports the hypothesis that MH obese may only represent a transition state to MU obese [10–12]. Fortunately, this transition may be reversible with adequate treatment, as it has been described how recovering a healthy metabolic status has a deeper impact on the reduction of associated comorbidities than a reduction in the body mass index alone [13].

In the last years, a special focus has been set on sex-specific differences in obesity. It is well known that men tend to accumulate fat in visceral depots, which has been related to a higher risk of suffering adverse outcomes, while women preferentially expand their subcutaneous depots [14]. Hormones have a huge impact on the metabolism of both sexes, with testosterone increasing muscle mass and basal metabolic rate. At the same time, estrogens play an important role in adipocyte differentiation, lipolysis/lipogenesis, and insulin sensitivity [15]. Thus, it should not be surprising that men and women have different predispositions to suffer adverse outcomes, experience disease progression differently, and react differently to treatments [16]. These differences in the onset of obesity raise the question of whether obesity development should be considered a different entity in men and women. In addition, it is attractive to speculate that the transition from MH to MU could also present sexual dimorphism. In this regard, a recent longitudinal study has observed that female MU obese patients present half the chance of returning to MH obese after a 5% BMI reduction than males [12]. Thus, identifying sex-differential selection and gene expression is essential for understanding obesity development and related morbidity.

From a mechanistic point of view, the adipose tissue expandability hypothesis has been proposed as a unifying theory that may explain the presence of MH obese patients [17]. According to this hypothesis, there is an individual threshold regarding the amount of fat that can be safely stored within the subcutaneous white adipose tissue (WAT). Once surpassed, triglycerides would begin to accumulate in ectopic tissues, such as visceral WAT (vWAT), liver, heart, kidneys, or skeletal muscle, exerting lipotoxic effects that compromise the functionality of these organs through oxidative stress, fibrosis, and inflammation [18].

Therefore, in this study, we aimed to compare the transcriptomic profiles of vWAT between MH and MU obese individuals and investigate potential sex-specific differences in these profiles. We hypothesized that the transcriptomic profiles of vWAT would differ between MH and MU obese individuals and that these differences would be influenced by sex. Understanding the molecular mechanisms controlling the transition from MH to MU and the potential sex-specific differences could pave the way for more personalized therapeutic strategies in the management of obesity and its associated metabolic disorders.

## Material and methods

### Study population

This monocentric study was carried out on 60 patients with morbid obesity, males and females ranging from 20–65 years old, attending the Unit of Morbid Obesity at the Hospital Universitario de Getafe for bariatric surgery from April 2018 to May 2019. This protocol has been approved by the Hospital Universitario de Getafe Ethical Committee (CEIm 18/30) and carried out following the Declaration of Helsinki principles (Code of Ethics of the World Medical Association) for experiments involving humans. All study participants signed informed consent after being informed about the purpose of this research protocol.

MU obesity was diagnosed based on the International Diabetes Federation criteria [7], with the presence of central obesity, defined as waist circumference [WC] ≥ 94 cm for males or WC ≥ 80 cm for females (if the BMI > 30 kg/m^2^ central obesity can be assumed); and at least one of the following: (a) hypertriglyceridemia, defined as triglyceride [TG] ≥ 1.7 mmol/L (150 mg/dL) or taking lowering TG drugs hypertension; (b) dyslipidemia, defined as high-density lipoprotein cholesterol [HDL-C] less than 1.03 mmol/L (40 mg/dL) for males or less than 1.29 mmol/L (50 mg/dL) for females, and or taking elevating HDL-C drugs; (c) raised blood pressure, with a measured systolic blood pressure [SBP] ≥ 130 mmHg or diastolic blood pressure [DBP] ≥ 85 mmHg, and/or taking antihypertensive drugs; (d) hyperglycemia, defined as fasting plasma glucose ≥ 5.6 mmol/L (100 mg/dL) or previously diagnosed type 2 diabetes. Participants with one of the previous metabolic abnormalities were categorized into the MU group, while participants with none of the above-mentioned abnormalities were categorized into the MH group.

### Anthropometrical evaluation, biochemical variables, and adipose tissue collection

One week before the elective surgery, anthropometrical evaluation included age, weight, and height measurements to calculate the BMI and waist-hip ratio. Blood samples were extracted after overnight fasting in ethylenediaminetetraacetic acid tubes, which were separated into plasma and serum aliquots by centrifugation (3500 rpm, 4 °C, 15 min) and then stored at − 80 °C until processing. A full biochemical analysis, including glucose and lipid profile, was performed using a conventional automated analyzer.

During the surgery, a portion of approximately 2 × 2 cm of vWAT was recovered and immediately snap-frozen in liquid nitrogen and stored at − 80 °C until processing.

### Adipose tissue histological analysis

vWAT was fixed in 4% paraformaldehyde for 48 h and embedded in paraffin. Slices (3 µm) were stained with hematoxylin and eosin. The stained slides were scanned with the Leica Aperio Versa system and analyzed with the Leica Aperio ImageScope 12.4. Snapshots were taken (Leica DFC495 Camera) using Leica Application Suite X software. The area of the adipocytes was analyzed using the Adiposoft plugin [19] in Fiji software [20].

### Microarray data and identification of differentially expressed transcripts

RNA was extracted from frozen vWAT using the RNeasy Mini Kit (Qiagen, Hilden, Germany) with DNase treatment following manufacturer procedure. RNA concentration was determined using a NanoDrop™ 2000/2000c Spectrophotometer, and its quality was assessed with Experion RNA StdSens analysis kit (Bio-Rad Laboratories Inc., Hercules, CA, USA), establishing an RQI ≥ 7 as selection criteria for the microarray experiments.

The gene expression profile of the samples was analyzed using GeneChip Human Gene 2.1 ST strips (Affymetrix, Thermo Fisher Scientific Santa Clara, California, USA). Following manufacturer instructions, 100 ng of RNA from each sample were mixed with Poly-A RNA and hybridized using GeneChip™ WT PLUS Reagent Kit. Then, double-strand cDNA was synthesized from the starting RNA, and cRNA was obtained by in vitro transcription of the double-stranded cDNA. Next, cRNA was used as a template of sense-strand cDNA by reverse transcription, which was subsequently fragmented by UDG and APE1 and labeled by TdT. Finally, the fragmented and labeled cDNA of all the samples was hybridized (4 samples for each strip) for 20 h at 48ºC, and after that, the strips were washed, stained, and imaged using the GeneAtlas System (Thermo Fisher Scientific).

CEL raw files were normalized and transformed into expression measures using Expression Console Software (Affymetrix). The Robust Multi-array Average (RMA) algorithm was used for background correction, normalization, and summarization of the probe-set level expression of the samples.

### qPCR validation

RNA was extracted and quantified using the same procedure as in the microarray. Reverse transcription was performed on 500 ng of RNA with PrimeScript RT MasterMix (Takara Bio, California, USA) using a combination of random hexamer primers and oligod(T). Optimal annealing temperature and amplicon sizes were checked for each pair of primers. RT-qPCR analyses were performed in a Quantstudio 5 Instrument (Applied Biosystems). 6,25 ng of cDNA were run in duplicate, and the mRNA levels were determined using intron-skipping primers, *ACTIN* as a housekeeping gene (forward 5´ CACCATTGGCAATGAGCGGTTC; reverse 5´ AGGTCTTTGCGGATGTCCACGT) and TB Green® Premix Ex Taq™ II (Tli RNaseH Plus) (Takara Bio). The genes used for validation were *FMO3 (*forward 5´ AACTCAGCCGCACAGCAGAA; reverse 5´ TCGAGTGACGAGCAGCATGT), *EGFL6 (*forward 5´ TGCAAGGCATCACGGGTTGT; reverse 5´ CCAGGTTCGCATGTAGCTTCACA), *ANGPT1 (*forward 5´ ACACGTGGAACCGGATTTCTCT; reverse 5´ TCTGGGCCATCTCCGACTTC) and *JUN (*forward 5´ AATCCAGTCCAGCAACGGGC; reverse 5´ TCACGTTCTTGGGGCACAGG).

### Bioinformatic analysis

The transcriptomic expression pattern of each group was analyzed by Transcriptome Analysis Console (TAC) software, considering a fold change higher and lower than 1.5 and −1.5, respectively, and p-value < 0.05, as cut-off criteria.

Functional enrichment of differentially expressed transcripts (DETs) was performed using Ingenuity Pathway Analysis (IPA; Qiagen). Following the identification of common components, further enrichment analysis was performed using FunRich software (http://www.funrich. org/). The KEGG, GO, Uniprot, Reactome, and FunRich databases were used to identify biological processes.

### Statistical analysis

Clinical and experimental data are expressed as mean ± SEM. The outliers were identified through the Rout method, using a Q = 1%. The normal distribution of each variable was verified with the Shapiro–Wilk and Kolmogorov–Smirnov tests. Statistical differences (p < 0.05) between MH and MU groups were assessed using a 2-tailed *t*-test for variables following a Gaussian distribution. Otherwise, a Mann–Whitney test was used. For those comparisons involving two variables, two-way ANOVA (2-ANOVA) followed by Tukey´s multiple comparison test was used. The statistical analyses were performed using GraphPad Prism (version 10.0) software (San Diego, USA). For the correlation analysis, Spearman correlation (ρ) between adipocyte size, clinical, biochemical and gene expression parameters was carried out using SPSS software.

## Results

### Participant characteristics

**Fig. 1:**
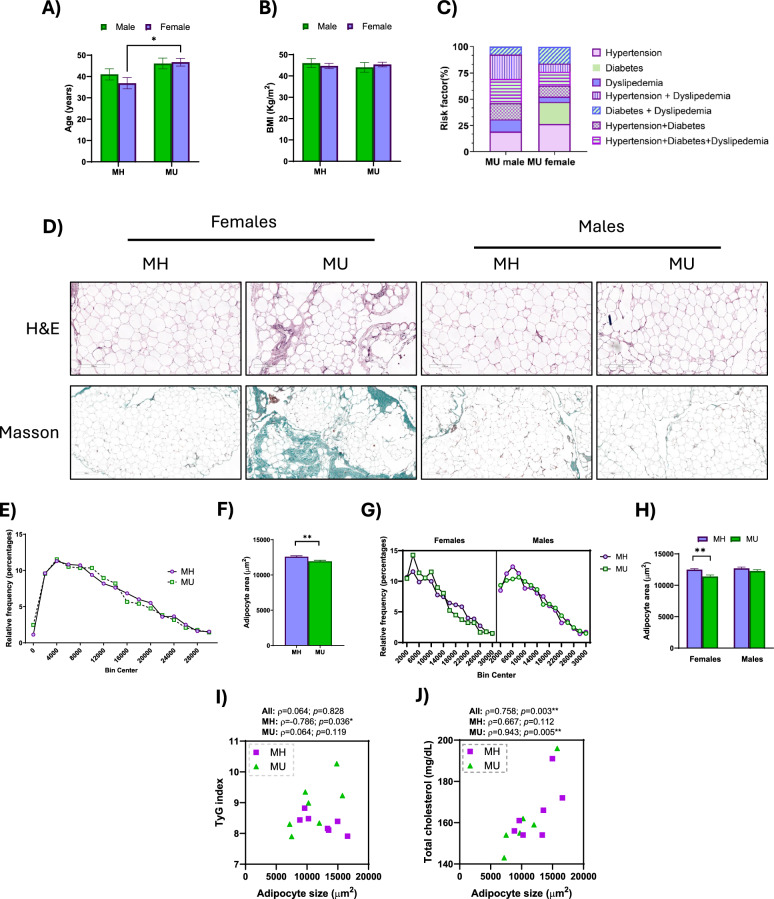
Characteristics of the patients. Clinical variables of the study population: **(A)** Age (years); **(B)** Body Mass Index (BMI; kg/m^2^), represented as the mean ± SEM. **(C)** Risk factors found for individuals in MU male and female groups. Histological analysis in 3 μm paraffin sections of vWAT obtained during the bariatric surgery: **(D)** Hematoxylin–eosin staining (above) and Masson trichrome staining (below). **(E)** Frequency distribution of adipocyte cell surface area in MH patients *vs.* MU patients. n = 6 per group. > 250 cells were measured for each patient. **(F)** Average adipocyte area in μm2. **(G)** Frequency distribution of adipocyte cell surface area stratified by sex. **(H)** Average adipocyte area in μm^2^. Results are represented as mean + SEM. Spearman correlation (ρ) between the triglyceride-glucose index **(I)** and total cholesterol **(J)**, and adipocyte size in the complete cohort, MH and MU patients. Differences were considered statistically significant at p < 0.05.* p < 0.05; ** p < 0.01

A graphical representation of the general characteristics registered for the study population of 60 individuals is shown in Fig. 1 (and extended in Table 1). MU patients were older on average than MH patients. However, when separated by sex, we only observed that difference in female patients since there were no statistical differences in the age of males. No statistical difference in BMI between MH and MU patients was observed (Fig. 1A-B). The distribution of the risk factors that categorized our patients as MU presented the following prevalence (Fig. 1C): hypertension (males: 19.2%, females: 26.3%), diabetes (females: 21.1%), dyslipidemia (males: 11.5%, females: 5.0%), hypertension and diabetes (males: 15.4%, females: 10.5%), hypertension, diabetes and dyslipidemia (males: 23.1%, females: 13.2%), hypertension and dyslipidemia (males: 23.1%, females: 7.9%), and diabetes and dyslipidemia (males: 7.7%, females: 15.8%).

**Table 1:** Population description and biochemistry panel

	MH	MU	MH		MU	
	All (n = 23)	All (n = 37)	Male (n = 9)	Female (n = 14)	Male (n = 11)	Female (n = 26)
Population description						
Age (years)	38.48 ± 1.92	46.54 ± 1.48 **	41.00 ± 2.65	36.86 ± 2.65	46.09 ± 2.54	46.73 ± 1.84 ^$^
BMI (kg/m^2^)	45.21 ± 1.09	44.99 ± 1.02	46.01 ± 2.12	44.70 ± 1.21	44.06 ± 2.27	45.38 ± 1.11
Glucose homeostasis						
Glucose (mg/dL)	94.04 ± 2.23	100.3 ± 1.95 *	98.56 ± 4.91	91.14 ± 1.62	99.72 ± 4.26	100.58 ± 2.13
Insulin (mIU/L)	19.59 ± 1.87	21.42 ± 1.77	23.99 ± 3.75	16.54 ± 1.41	23.09 ± 2.75	20.76 ± 2.24
HOMA-IR index	4.63 ± 0.50	5.14 ± 0.40	5.92 ± 1.02	3.73 ± 0.30	5.78 ± 0.76	4.87 ± 0.46
HOMA-B index	238.70 ± 23.54	204.4 ± 18.01	266.89 ± 41.34	219.23 ± 27.85	247.80 ± 39.64	187.00 ± 19.07
HbA1c (IFCC; mmol/mol A1c)	34.06 ± 0.85	35.12 ± 0.9	34.88 ± 1.45	33.25 ± 0.90	34.44 ± 1.57	35.47 ± 1.12
Lipid profile						
Triglycerides (mg/dL)	104.9 ± 6.81	132.2 ± 8.44 *	107.89 ± 15.28	103.00 ± 5.98	137.55 ± 12.31	129.80 ± 11.00
Total cholesterol (mg/dL)	167.2 ± 6.26	175.9 ± 5.25	166.22 ± 7.76	167.79 ± 9.22	167.73 ± 9.44	179.35 ± 6.31
HDL-cholesterol (mg/dL)	45.50 ± 21.12	45.33 ± 1.45	48.13 ± 4.12	43.75 ± 2.24	42.44 ± 2.38	46.57 ± 1.77
LDL-cholesterol (mg/dL)	103.7 ± 6.49	103.5 ± 5.00	99.50 ± 6.04	106.50 ± 10.20	95.30 ± 7.84	107.00 ± 6.27
VLDL-cholesterol (mg/dL)	20.74 ± 2.46	28.11 ± 1.88 *	22.80 ± 4.74	20.55 ± 1.58	30.82 ± 2.76	26.87 ± 2.42
Kidney function						
Creatinine (mg/dL)	0.83 ± 0.03	0.77 ± 0.03	0.98 ± 0.05	0.74 ± 0.02 ^+++^	0.85 ± 0.02	0.74 ± 0.03 ^+++^
Albumin (g/dL)	4.46 ± 0.06	4.59 ± 0.05	4.56 ± 0.06	4.40 ± 0.09	4.78 ± 0.09	4.50 ± 0.05 ^+^
Ions						
Na^+^ (mmol/L)	141.00 ± 0.41	141.20 ± 0.29	141.22 ± 0.78	140.93 ± 0.49	141.91 ± 0.42	140.89 ± 0.36
K^+^ (mmol/L)	4.44 ± 0.06	4.39 ± 0.05	4.45 ± 0.11	4.43 ± 0.07	4.48 ± 0.10	4.35 ± 0.06
Cl^−^ (mmol/L)	104.2 ± 0.45	103.2 ± 0.43	103.89 ± 0.56	104.43 ± 0.65	103.18 ± 0.63	103.27 ± 0.56
Ca^2+^ (mmol/L)	9.40 ± 0.06	9.44 ± 0.06	9.50 ± 0.10	9.34 ± 0.08	9.50 ± 0.15	9.44 ± 0.07
P^+^ (mmol/L)	3.09 ± 0.11	3.42 ± 0.08 *	3.22 ± 0.18	3.00 ± 0.14	3.16 ± 0.16	3.53 ± 0.09 ^$^
Mg^2+^ (mmol/L)	2.02 ± 0.03	2.01 ± 0.03	2.02 ± 0.06	2.03 ± 0.04	2.02 ± 0.05	2.00 ± 0.04
Liver function						
GPT (U/L)	18.55 ± 1.34	22.47 ± 1.81	20.29 ± 2.12	17.62 ± 1.72	31.1 ± 3.2^$$^	19.15 ± 1.84^++^
GGT (U/L)	19.95 ± 1.72	23.13 ± 2.17	24.63 ± 2.72	17.29 ± 1.93	28.11 ± 4.31	20.43 ± 2.23
ALP (U/L)	77.52 ± 4.39	71.19 ± 2.29	74.78 ± 7.26	79.29 ± 5.66	71.09 ± 4.80	71.23 ± 2.62
LDH (U/L)	183.70 ± 7.17	181,90 ± 5.32	188.63 ± 10.76	180.93 ± 9.65	186.27 ± 8.80	180.04 ± 6.66
Iron metabolism						
Iron (µg/dL)	73.56 ± 7.02	75.71 ± 4.92	80.78 ± 12.43	66.35 ± 6.46	85.32 ± 8.95	71.78 ± 5.81
Ferritin (ng/mL)	122.70 ± 24.02	97.08 ± 14.30	151.47 ± 37.80	97.48 ± 29.96	171.83 ± 28.96	69.91 ± 12.30^++^
Transferrin (ng/mL)	258.80 ± 9.80	270.00 ± 6.34	253.31 ± 17.44	263.59 ± 11.19	270.22 ± 13.82	269.89 ± 7.11
Total iron binding capacity (µg/dL)	360.10 ± 13.86	377.40 ± 10.35	357.76 ± 24.74	362.40 ± 14.77	381.08 ± 19.49	375.78 ± 12.51
Transferrin saturation (%)	21.73 ± 2.09	20.39 ± 1.50	26.29 ± 3.09	17.18 ± 1.56	22.57 ± 2.41	19.46 ± 1.88
Vitamins						
Folate (ng/mL)	4.30 ± 0.41	5.28 ± 0.47	4.20 ± 0.70	4.40 ± 0.49	5.01 ± 0.70	5.39 ± 0.60
Vit B12 (pg/mL)	332.70 ± 34.80	390.80 ± 24.87	392.86 ± 59.96	280.00 ± 31.54	427.11 ± 37.83	373.53 ± 31.85
Vit D (ng/mL)	22.40 ± 2.16	21.14 ± 2.25	25.57 ± 2.98	19.63 ± 2.92	24.78 ± 3.39	19.50 ± 2.86
Hormones						
TSH (µU/mL)	2.87 ± 0.55	2.07 ± 0.43	3.48 ± 0.80	2.09 ± 0.62	1.71 ± 0.88	2.23 ± 0.52
Cortisol (µg/dL)	9.81 ± 0.66	11.94 ± 0.61 *	10.56 ± 1.19	9.13 ± 0.67	12.80 ± 1.18	11.63 ± 0.71
PTH (pg/mL)						
Inflammation						
C-reactive Protein (mg/L)	6.58 ± 0.79	5.72 ± 0.62	5.23 ± 1.10	7.48 ± 1.05	4.31 ± 0.79	6.27 ± 0.78
Sedimentation velocity (mm/h)	11.94 ± 1.64	19.48 ± 2.31 *	10.44 ± 2.19	13.44 ± 2.46	8.13 ± 0.95	23.44 ± 2.63 ^++^
Coagulation						
INR	1.01 ± 0.008	1.01 ± 0.008	1.17 ± 0.15	1.00 ± 0.008	1.02 ± 0.01	1.00 ± 0.01
Prothrombin time (s)	99.59 ± 1.88	100.6 ± 1.78	95.45 ± 3.21	101.96 ± 2.16	95.71 ± 2.95	102.41 ± 2.11
Activated Partial Thromboplastin Time (s)	28.09 ± 0.48	28.19 ± 0.32	29.19 ± 1.08	28.34 ± 0.71	28.84 ± 0.65	27.95 ± 0.37

HOMA-IR (Glucose (mmol/l) x Insulin (mIU/L)/22.5

HOMA-B (20 x Insulin (miU/L)/(Glucose (mmol/L) - 3.5

*p<0.05, **p<0.01 MH vs MU (all patients included)

+p<0.05, ++p<0.01 Female vs male

^$^p<0.05 MU vs MH

Prior to the elective bariatric surgery, a full biochemistry panel was performed on every patient (Table 1). The cohort of MU patients presented higher glucose, triglycerides, VLDL-Cholesterol, and cortisol circulating levels before the surgery in comparison to the MH patients. However, when they were stratified by sex, no differences were observed in any of these metabolic parameters. MU patients also exhibited higher levels of P^+^ and sedimentation velocity than the MH cohort. The separation by sex revealed that these differences were specific to the female group since no differences were observed between the MU and MH males.

Finally, certain parameters did not exhibit differences according to the metabolic state when both males and females were taken together, but shown statistical differences when the sex-dependent stratification was carried out. That is the case of creatinine, albumin, GPT and ferritin. Creatinine levels were lower in MU males and females compared to their MH counterparts. Circulating albumin, GPT, and ferritin were lower in the female MU compared to the MH females, but no differences were observed in males.

For the study of the transcriptome of the vWAT, 16 patients were randomly selected: 8 MH (4 males and 4 females) and 8 MU (4 males and 4 females). The biochemical parameters of this subset of patients are summarized in the Supplementary Table 1. We only found significant differences in age and in the GGT levels between MH and MU patients. However, when they were separated by sex, these differences were no longer significant.

During the surgery, a portion of vWAT was excised and fixed in formaldehyde, and a histological analysis was performed to analyze adipocyte size distribution and collagen deposition (Fig. 1D). The vWAT of MU females presented a marked fibrosis in relation with the other groups. When analyzed globally, the average size of the adipocytes within the vWAT of MU patients had a lower area than those from the MH patients. They presented a higher frequency of adipocytes in the range of 10,000–14,000 µm^2^, but those in the range of 16,000–20,000 µm^2^ were less abundant (Fig. 1E-F). However, when both groups were analyzed by sex, we observed that these differences were due to the MU female group, which exhibited a higher frequency of small adipocytes than the MH females, that was reflected in a reduced average size. However, no differences were found in the distribution and average size of the adipocytes of MU males *vs* MH males (Fig. 1G-H).

The size of the adipocytes inversely correlated with the triglyceride/glucose (TyG) index (ρ =—0.786; p = 0.036), a parameter that has been proposed as a novel marker of cardiometabolic risk, in the MH patients (Fig. 1I). In addition, adipocyte size positively correlated with total cholesterol in the full cohort (ρ = 0.758; p = 0.003), but thanks to the contribution of the MU patients (ρ = 0.943; p = 0.005), since no significant correlation was found with the MH patients (Fig. 1J).

### Identification of sex-specific DETs in MH and MU obese individuals

**Fig. 2:**
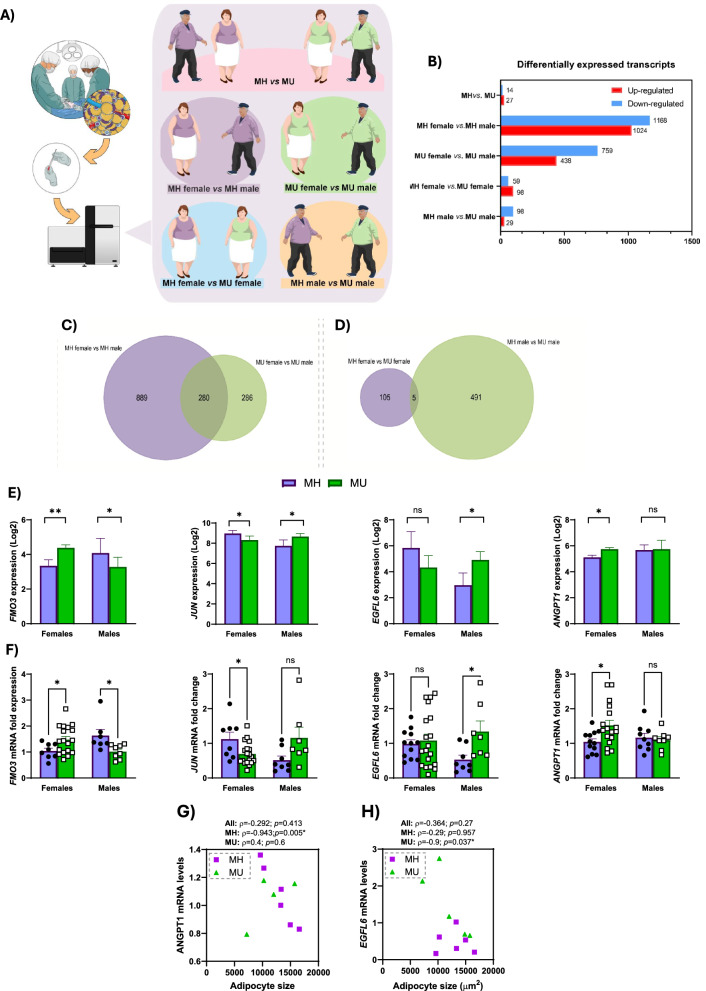
Profiles of differentially expressed mRNAs in vWAT from the diverse groups. (**A**) Flowchart of the study population and the protocol of the experimental steps, including transcriptomic analysis. **(B)** DETs obtained in each group. The colors indicate the expression level ranging from blue (downregulated) to red (upregulated). **(C)** Venn diagram of differentially expressed mRNAs between MH female *vs.* MH male compared to MU female *vs.* MU male, and **(D)** MH female *vs*. MU female compared to MH male *vs*. MU male. **(E)** Bar graphs representing DETs overlapping between groups in the 16 patients included in the array. **(F)** qPCR validation in vWAT samples from 48 patients. Results are represented as mean + SEM. * p < 0.05; ** p < 0.01. Spearman correlation (ρ) between *EGFL6* **(G)** and *ANGPT1* **(H)** mRNA levels, and adipocyte size in the complete cohort, MH and MU patients. Differences were considered statistically significant at p < 0.05

To elucidate the potential sex-specific alterations in the transcriptome of MH and MU obese individuals, vWAT samples were obtained from this study population from 4 patients of each group. Figure 2A shows a schematic representation of the study workflow. The differential transcriptome profiles in the diverse groups were obtained when a 1.5-fold change and *p*-value < 0.05 served as cut-off criteria (Fig. 2B). 41 DETs were identified in the MH *vs.* MU group (27 upregulated and 14 downregulated); 2,192 DETs were identified in the MH female *vs.* MH male group (1,024 upregulated and 1,168 downregulated); 1,197 DETs were identified in the MU female *vs.* MU male group (438 upregulated and 759 downregulated); 157 DETs were identified in the MH female *vs.* MU female group (98 upregulated and 59 downregulated); and 127 DETs were identified in the MH male *vs.* MU male group (29 upregulated and 98 downregulated).

These results included coding transcripts as well as long-intergenic non-coding transcripts, so the database was curated to include exclusively identified mRNA sequences. Surprisingly, we only found 14 downregulated and 18 upregulated mRNAs between these 2 groups. However, when we separated our patients by sex, larger differences were observed. MH female presented 889 exclusively differentially expressed mRNAs in comparison to MH male. Likewise, MU female showed 286 exclusively differentially expressed mRNAs compared to MU male. Venn diagram analysis revealed 280 common transcripts between MH female *vs.* MH male and MU female *vs.* MU male (Fig. 2C). However, of these 280 common transcripts, only two transcripts demonstrated a different expression pattern between these two groups: flavin-containing dimethylaniline monooxygenase 3 (*FMO3*) and Plasmolipin (*PLLP)*. The vWAT in the transition from MH to MU also showed a differential expression profile between men and women. 105 exclusively mRNAs were differentially expressed between MH females and MU females. On the other side, the bioinformatic analysis revealed 491 differentially exclusively expressed mRNAs between MH males and MU males. An integrated comparison between the MH female *vs.* MU female and MH male *vs.* MU male groups by Venn diagram analysis identified 5 common transcripts (Fig. 2D). Of these 5 transcripts, only two showed a diverse expression pattern: *FMO3* and *JUN* gene transcripts, showing a sex-specific differential expression profile (Fig. 2E).

To validate our results, the expression of these two genes, together with *ANGPT1* and *EGFL6* was measured by qPCR in the vWAT samples of 48 individuals from our cohort (Fig. 2F). We found that the mRNA levels of the 4 genes measured by qPCR resembled the results obtained in the transcriptome array: *FMO3* and *JUN* showed a sex-specific expression pattern; EGFL6 expression was increased in MU males in comparison to MH males and *ANGPT1* mRNA levels were only increased in MH females in comparison to MH females.

In fact, adipocyte size inversely correlated with the mRNA levels of *ANGPT1* in the MH patients (ρ =—0.943; p = 0.005; Fig. 2G) and with the mRNA levels of *EFGL6,* but only in the MU patients (ρ =—0.9; p = 0.005; Fig. 2H).

### Sex-specific differences in the vWAT transcriptome in obesity

**Fig. 3:**
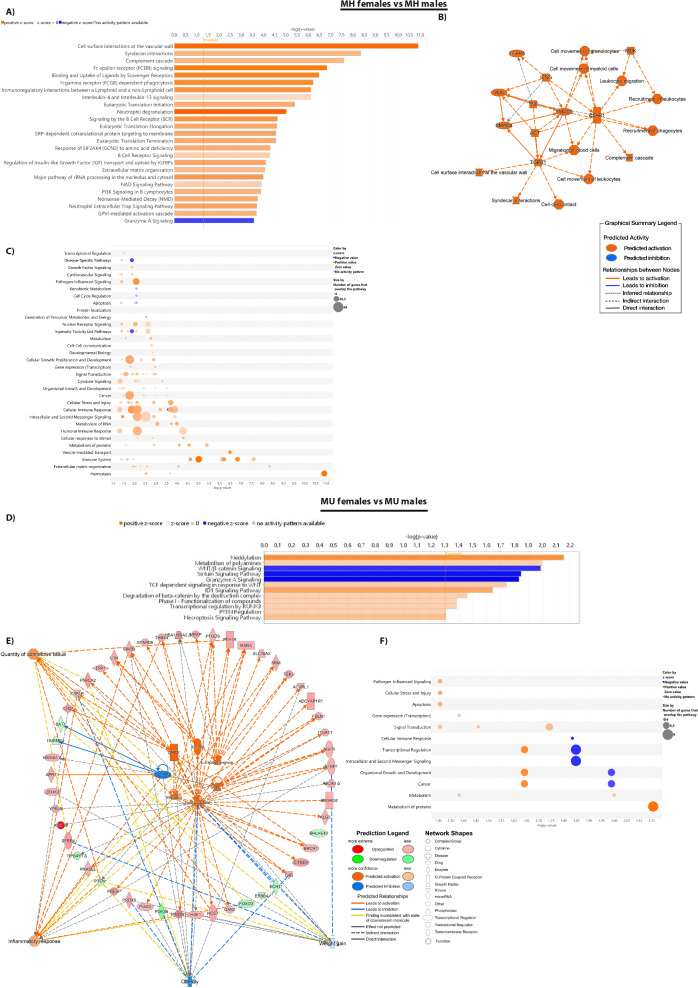
Sex-differences in the transcriptome of vWAT from patients with obesity and different metabolic profile.** (A)** Ingenuity pathway analysis (IPA) top-25 canonical pathways, listed according to their *p* value (− log*).* **(B)** Graphical summary of the major biological themes including canonical pathways, upstream regulators, diseases, and biological factors. **(C)** Bubble chart representing the pathway category, listed according to their -log(*p* value). **(D)** Ingenuity pathway analysis (IPA) canonical pathways, listed according to their *p* value (− log; orange line). **(E)** Network analysis of the upstream regulators, targeted DETs and diseases and functions. **(F)** Bubble chart representing the pathway category, listed according to their -log(*p* value)

To dissect the signaling pathways that characterize the differences between female and male patients, we performed a Canonical Pathway Analysis with the IPA software. Based on IPA algorithms, and considering a -log(p value) = 1.30 and |Z-score |> 1, the 1,082 differentially expressed and annotated mRNAs between female and male MH patients (787 upregulated and 295 downregulated) were enriched in 104 canonical pathways (Supplemental Table S2). The top-25 canonical pathways ordered by p-value are shown in Fig. 3A. IPA analysis predicts an upregulation in the adipose tissue of MH females in canonical pathways such as surface interaction at the vascular wall, Fc Epsilon and Fc Gamma receptor signaling, phagocytosis and neutrophil degranulation, B-cell receptor signaling, IGF transport and uptake by IGFBP and protein translation pathways. The only downregulated pathway was Granzyme A signaling, suggesting a defective cytotoxic capacity to eliminate damaged cells within the adipose tissue.

The network analysis has identified several key modulators of immune and inflammatory responses, such as Complement C5a receptor (*C5AR1*), *TNFA, IFNG, JUN, AGT, MDK* and fibrotic responses, mainly through the TGFB1 pathway (via SMAD3/4 signaling) (Fig. 3B**).** Taken together, the adipose tissue of female MH patients presented an overactivation of homeostasis-related pathways, as well as extracellular matrix (ECM) organization, immune system and cytokine signaling, cellular stress and injury in comparison to male MH patients (Fig. 3C).

Once the pathological obesity has been established, the transcriptome analysis revealed a differential expression in 424 annotated DETs (125 downregulated and 299 upregulated) between MU females and males. The IPA analysis revealed that these DETs were significantly enriched in 12 canonical pathways (Supplemental Table S3), including an upregulation in neddylation, metabolism of polyamines, TCF dependent signaling in response to WNT, ID1 signaling pathway, degradation of beta-catenin, transcriptional regulation by Runx3, PTEN regulation, necroptosis signaling pathway. On the contrary, we found a downregulation in the WNT/beta-catenin, sirtuin and granzyme A signaling pathways (Fig. 3D).

When comparing MU females with MU males, the proposed upstream regulators include the increase in sexual hormones (beta-estradiol, estrogen), *IL10RA, SORL1, DRD2*, L-triiodothyronine and a reduction in *COPS5*. Overall, the activation network predicts the activation of cellular processes such as inflammatory response and quantity of connective tissue, but a predicted inhibition on lipid-accumulation pathologies such as weight gain and obesity (Fig. 3E). The differential activity of these canonical pathways affects cellular processes such as general and protein metabolism, growth and development and signal transduction (Fig. 3F).

### Changes in the vWAT transcriptome in the transition from MH to MU obesity

**Fig. 4:**
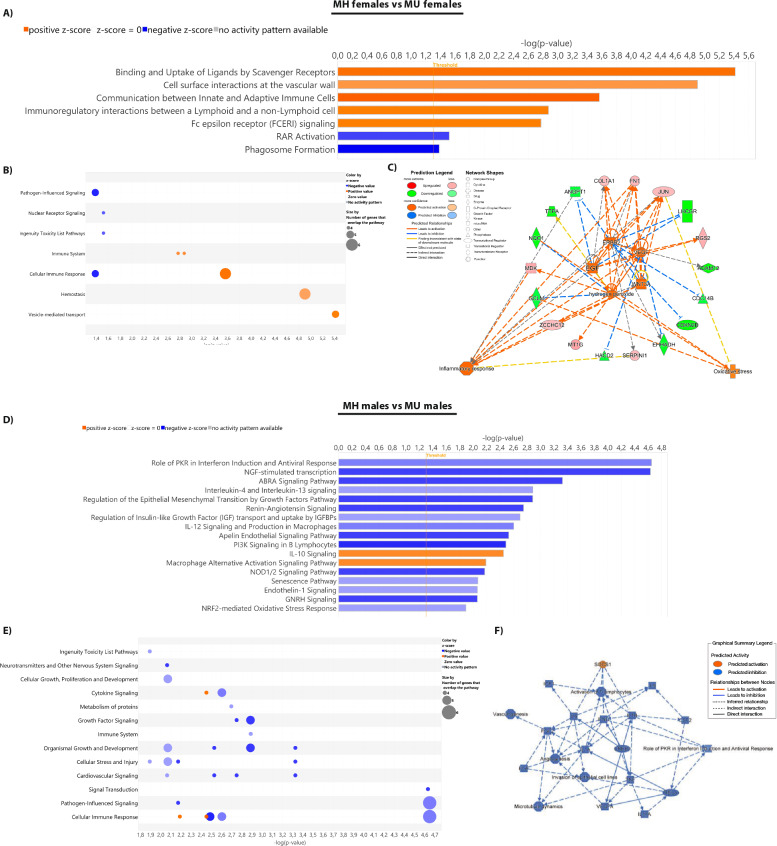
Differential transition from MH to MU obesity in females and males according to the vWAT transcriptome. **(A)** Ingenuity pathway analysis (IPA) canonical pathways, listed according to their *p* value (− log; orange line*).* **(B)** Bubble chart representing the pathway category, listed according to their -log(*p* value). **(C)** Network analysis of the upstream regulators, targeted DETs and diseases and functions. **(D)** Ingenuity pathway analysis (IPA) canonical pathways, listed according to their *p* value (− log; orange line). **(E)** Bubble chart representing the pathway category, listed according to their -log(*p* value). **(F)** Graphical summary of the major biological themes including canonical pathways, upstream regulators, diseases, and biological factors

To get insight into the mechanisms that define the transition from MH obesity to MU obesity in females, we analyzed the 87 annotated mRNAs after curation when we compared MH females with MU females. These DETs were enriched in 7 canonical pathways (Fig. 4Aand Supplemental Table S4), where the IPA analysis predicts an upregulation in binding and uptake of ligands by scavenger receptors, cell surface interaction at the vascular wall, communication between innate and adaptive immune cells, immunoregulatory interactions between a lymphoid and a non-lymphoid cell, Fc epsilon receptor signaling and a reduction in retinoic acid receptor (RAR) activation and phagosome formation, suggesting changes in vesicle-mediated transport, hemostasis and immune response (Fig. 4B). Overall, the activation network predicts an activation of cellular processes such as inflammatory response and oxidative stress, mediated by upstream regulators such as hydrogen peroxide, *EGF*, *VEGF, ERBB2* and *WNT3A* in the MH females (Fig. 4C).

However, in males, the transition from MH to MU obesity revealed more differences in the transcriptome of the adipose tissue. The total number of DETs was similar to what we found in females (127 total DETs, reduced to 87 after curation; 73 downregulated and 14 upregulated). These transcripts were enriched in several canonical pathways (Fig. 4D and Supplemental Table S5) that were predicted to be downregulated, including the role of PKR on interferon induction and antiviral response, NGF-stimulated transcription, ABRA signaling pathway, interleukin 4 and 13 signaling, renin-angiotensin signaling, regulation of IGF transport and uptake by IGFBP, IL-12 signaling in macrophages, apelin endothelial signaling pathway, PI3K signaling in B lymphocytes, NOD1/2 signaling pathway, senescence pathway, endothelin-1 pathway, GNRH pathway, NRF-2 mediated response to oxidative stress. On the other hand, the IL-10 pathway and the macrophage alternative activation pathway were predicted to be upregulated in the white adipose tissue of MH male patients.

Taken together, these results suggest that MH male patients exhibit a reduced inflammatory response, as well as a reduced cellular stress (Fig. 4E). The network analysis of the potential key regulators indicate the inhibition of proinflammatory mediators, such as PKR, INF, IL12 and NOD1/2, stress inductors (NGF, ABRA, renin-angiotensin, IGF, among others) and the activation of anti-inflammatory mediators (IL-10, M2 alternative activation of macrophages) as potential responsible in the maintenance of an anti-inflammatory environment that contributes to keep the adipose tissue homeostasis in the obese, but MH male patients (Fig. 4F).

### Functional enrichment analysis of sex-specific DETs in MH and MU obese individuals

Considering that the main differences that our transcriptome analysis had revealed so far was mostly related to inflammation and ECM, we wanted to determine if the transition from MH to MU was similar in these processes between females and males.

**Fig. 5:**
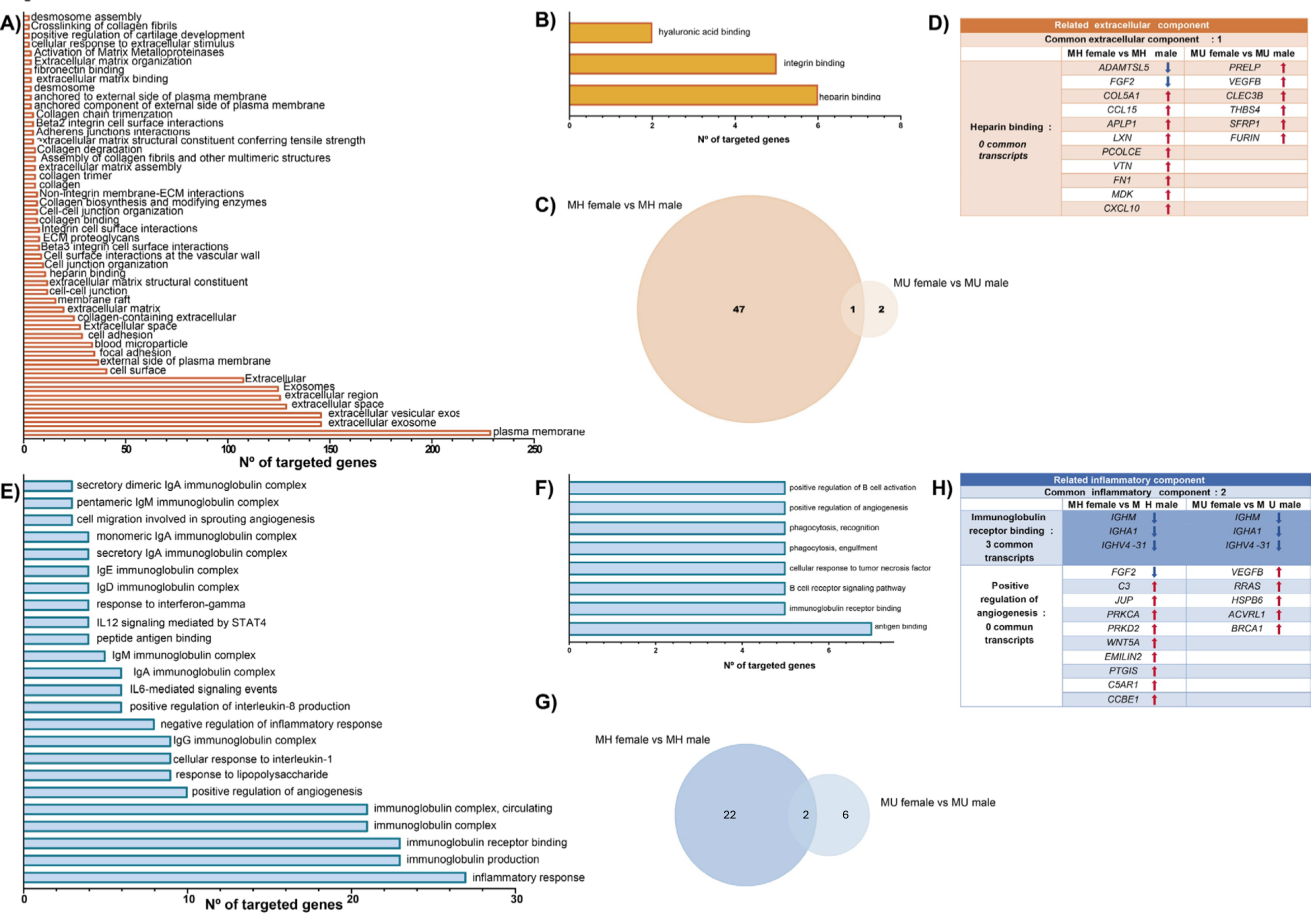
Functional enrichment analysis of differentially expressed mRNAs of MH female *vs.* MH male and MU female *vs.* MU male groups. Bar plots represent functional enrichment analysis of related extracellular components of MH female *vs*. MH male **(A)** and MU female *vs*. MU male **(B)**. **(C)** Venn diagram of a related extracellular component of MH female *vs.* MH male and MU female *vs*. MU male groups. **(D)** Common related extracellular components and their respective transcripts of MH female *vs*. MH male and MU female *vs*. MU male groups. Bar plots represent functional enrichment analysis of related inflammatory components of MH female *vs.* MH male (**E**) and MU female *vs*. MU male **(F)**. **(G)** Venn diagram of related inflammatory component of MH female *vs.* MH male and MU female *vs.* MU male groups. **(H)** Common related inflammatory components and their respective transcripts of MH female *vs.* MH male and MU female *vs.* MU male groups. The arrow colors indicate the expression level of the transcripts (red shows upregulation, and blue shows downregulation)

Transcripts found to have differential expression profiles of each group were analyzed for functional enrichment associated with GO, Uniprot, and Reactome databases. Functional enrichment of the MH female *vs.* MH male and MU female *vs.* MU male groups was performed (Fig. 5). The MH female *vs*. MH male group showed 46 extracellular-associated components that were significantly enriched (Fig. 5A and Supplementary Table S6.). However, the MU female *vs.* MU male analysis only presented 3 significantly enriched extracellular-associated components (Fig. 5B and Supplementary Table S7). The only one that was shared with the MH female *vs.* MH male group was the heparin-binding component (Fig. 5C). Nevertheless, the DETs of the heparin-binding component are completely different between MH female *vs.* MH male and MU female *vs.* MU male groups, as shown in Fig. 5D. Regarding the inflammatory-associated components, the MH female *vs.* MH male and MU female *vs.* MU male groups displayed 30 and 8 significantly enriched processes, respectively (Figs. 5E and F, Supplementary Table S8 and S9). Venn diagram analysis identified 2 shared inflammatory-associated components: immunoglobulin receptor binding and the positive regulation of angiogenesis (Fig. 5G). Although immunoglobulin receptor binding demonstrated three common transcripts between the groups (*IGHM, IGHA1,* and *IGHV4-31*), no different expression profile was observed (Fig. 5H). In contrast, no DETs were shared in the positive regulation of the angiogenesis-enriched component (Fig. 5H).

**Fig. 6:**
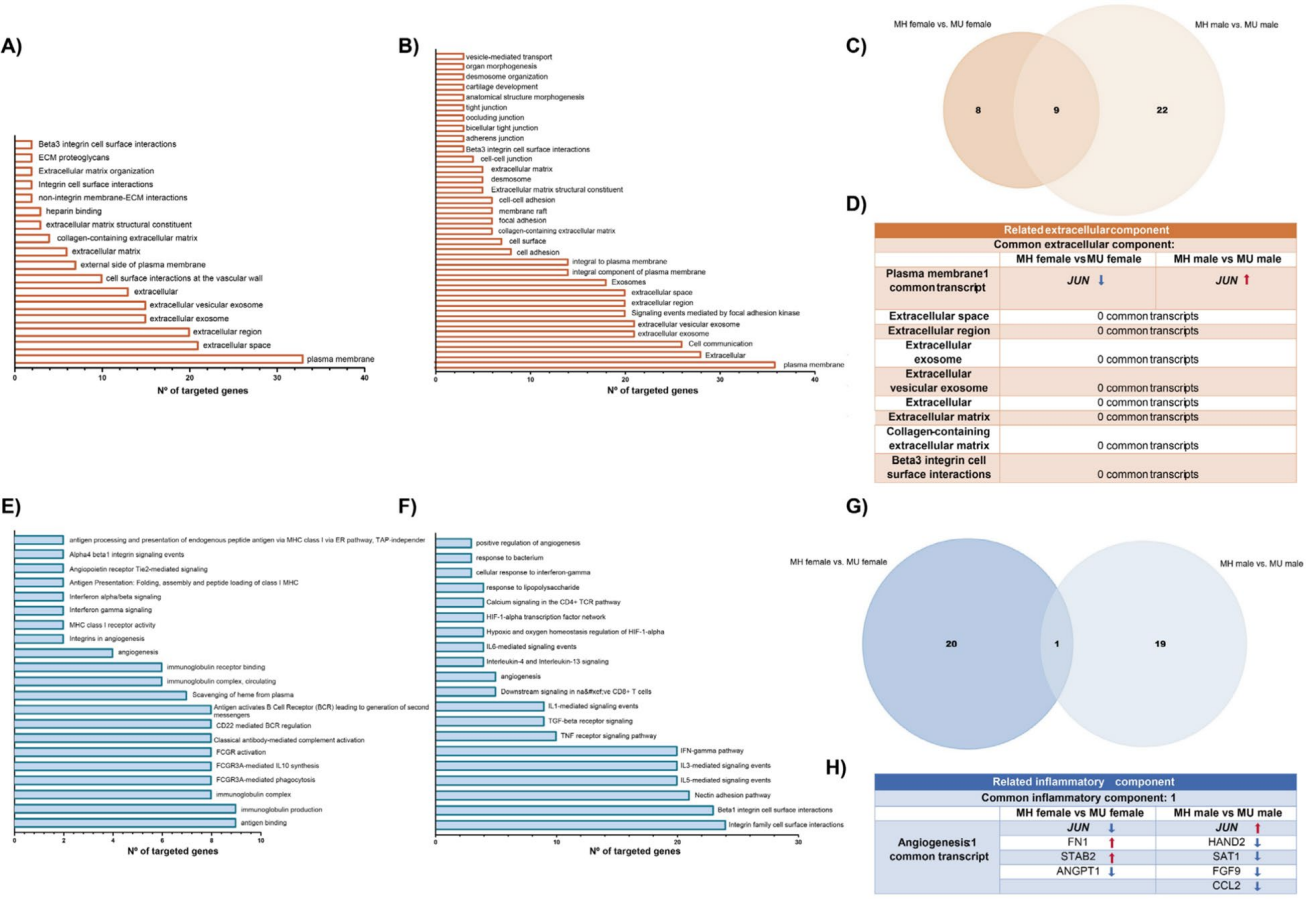
Functional enrichment analysis of differentially expressed mRNAs of MH female *vs*. MU female and MH male *vs*. MU male groups. Bar plots represent functional enrichment analysis of related extracellular components of MH female *vs.* MU female **(A)** and MH male *vs.* MU male **(B)**. **(C)** Venn diagram of a related extracellular component of MH female *vs.* MU female and MH male *vs.* MU male groups. **(D)** Common related extracellular components and their respective transcripts of MH female *vs*. MU female and MH male *vs*. MU male groups. Bar plots represent functional enrichment analysis of related inflammatory components of MH female *vs.* MU female **(E)** and MH male *vs.* MU male **(F)**. **(G)** Venn diagram of related inflammatory component of MH female *vs.* MU female and MH male *vs.* MU male groups. **(H)** Common related inflammatory components and their respective transcripts of MH female *vs*. MU female and MH male *vs.* MU male groups. The arrow colors indicate the expression level of the transcripts (red shows upregulation, and blue shows downregulation)

Then, the functional enrichment of MH and MU groups differentiated by sex was realized, as shown in Fig. 6. Regarding extracellular-related components, MH female *vs.* MU female and MH male *vs.* MU male groups presented 18 and 31 significantly enriched components, respectively (Figs. 6A and B). 9 of these extracellular-related components were shared between these groups: plasma membrane, extracellular space, extracellular region, extracellular exosome, extracellular vesicular exosome, ECM, collagen-containing ECM and beta3 integrin cell surface interactions (Fig. 6D and Supplementary Table S10). Nonetheless, only the plasma membrane-enriched component was shared between these groups (Fig. 6D and Supplementary Table S11). Interestingly, both groups presented a common transcript, *JUN*, whose expression varies according to the group (Fig. 6D). Concerning the inflammatory-related components, MH female *vs*. MU female and MH male *vs*. MU male groups displayed 23 and 20 components, exhibiting 1 common: angiogenesis (Fig. 6E-H). Again, *JUN* was the only common transcript of this pathway between these groups (Fig. 6G).

## Discussion

The concept of MH obesity is currently recognized as a temporary trait that will eventually lead to the apparition of cardiovascular comorbidities [21, 22]. Approximately 35% of MH patients with obesity transitioned to MU obesity in a follow-up period of 3.5 years [23], but this rate increased up to 60–70% after 7 years [24, 25]. Nonetheless, it is important to note that the transition from unhealthy to healthy obesity is also possible, as it has been observed in response to efficient health interventions [26]. Thus, from a clinical point of view, the identification of the obesity-related phenotype can be crucial to start early therapeutic approaches that will reduce the risk of suffering adverse outcomes, as the percentage of MH patients decreases with age independently of the BMI [27]. From a basic research point of view, the study of this population has increased the knowledge that we currently have in relation to the causes, pathological bases, and consequences of obesity, identifying the expansion of the adipose tissue as a key mediator in the physiopathology of this disease [28].

Considering that sexual hormones are involved in adipose tissue expansion, we wanted to investigate the sex-based differences that control the transition from one metabolic phenotype to another. To address this issue, we analyzed the transcriptome of vWAT from patients with morbid obesity and different metabolic profiles. The main outcome of this study is that the vWAT of female patients is more prone to develop a pathological profile in obesity, both in the presence and in the absence of comorbidities. Furthermore, the transition from a MH into a MU state also exhibits a different signature in both sexes. Taken together, our results support the necessity of treating obesity as a different entity in men and women.

When we analyzed the transcriptome of MH *vs.* MU patients prior to sex stratification, we found a similar profile between both groups. Only 41 DETs were found, and they were mostly related to immune response and glucose homeostasis. These results are in consonance with other reports in subcutaneous and vWAT [29, 30] and have contributed to establishing the connection between defective adipose tissue and increased cardiovascular risk.

However, the main novelty of this report is that when we stratified by sex, the number of DETs increased by at least 50-fold, revealing the differences between women and men. Surprisingly, the vWAT of MH females presented a strong proinflammatory profile, fibrosis, and oxidative stress compared to vWAT in the MH males. The upregulation of pathways related to neutrophil function, phagocytosis, Fc receptors and B cells activation, and the inhibition of the Granzyme A signaling suggest a defective elimination of dysfunctional adipocytes, which can further contribute to chronic inflammation. It is known that women rather expand their subcutaneous adipose tissue in obesity [14], so it is attractive to speculate that their vWAT is not as prepared to store energy efficiently as in men. In fact, these differences have been recently described in the vWAT from female obese mice [31], that presented more inflammation and T cell infiltration [32], in an estrogen dependent manner. Estrogens promote subcutaneous fat growth (with the corresponding cardioprotective advantages) but inhibit vWAT expansion [33]. On the contrary, estrogen deficiency could promote vWAT hypertrophy, decrease vWAT vascularization [34], promote vWAT immune activation, inflammation, and fibrosis [35]. In fact, the upstream analysis of the comparison between men and women has identified beta-estradiol (z-score: 5.691; p-value of overlap = 8.023 × 10^–23^) and estrogen (z-score: 2.699; p-value of overlap = 1.760 × 10^–9^) as key upstream activators.

These sex-specific differences are also evident when we compared MU women and men. More than 1200 DETs were found in the vWAT, and the enrichment analysis revealed that, in women, this tissue presents a higher remodeling and expansion. For instance, the increased neddylation pathway is necessary for adipogenesis, as its inhibition disrupts CREB/CEBPβ/PPARγ signaling [36] and ID1 regulates cell proliferation in vWAT [37]. This is supported by the histological analysis that revealed a higher frequency of small adipocytes in MU females than MU males. We also found increased inflammation and cell death (as inferred from increased levels of *TNFA*, necroptosis, *RUNX3*, and *PTEN*) and poor metabolic control (due to decreased sirtuin and WNT/ß-catenin pathways). The analysis of the upstream regulators may suggest that, despite the inflammatory process and connective tissue accumulation, there is an incomplete attempt to adapt vWAT in females, characterized by the activation of the anti-inflammatory *IL10RA* and modulation of energy and dopaminergic metabolism. In fact, the bioinformatic analysis predicted a reduction in biological functions and diseases related to fat accumulation in the vWAT of MU females *vs.* males, despite having similar BMIs.

Proper adipose tissue expansion requires remodeling of the ECM [38]. Adipocytes are enmeshed in an ECM network that provides structural support and mediates various signaling pathways [39]. The expansion of adipose tissue depends on adaptive cellular and extracellular adaptations to avoid harmful ectopic lipid accumulation and lipotoxicity [40–42]. However, when this expansion becomes dysfunctional, as often seen in obesity, it initiates a cascade of inflammatory events [43]. The relevance of the tissue expansion was also highlighted in the full cohort of patients via correlation analysis. We found that, in MH patients, allowing a better expansion of the tissue through the increase in the adipocyte size was inversely proportional to the TyG ratio, that has been described as a marker of cardiometabolic risk [44]. However, in MU patients, the size of the adipocytes correlates positively with the levels of circulating cholesterol.

Specifically, overloaded adipocytes and the resulting lipotoxicity drive chronic, low-grade inflammation, marked by macrophage infiltration and their polarization towards a pro-inflammatory (M1) phenotype [45]. This macrophage accumulation, driven by danger signals and inflammatory cues, contributes to fibrosis through excessive ECM deposition, further exacerbating inflammation and potentially leading to adipocyte necrosis [38, 45]. While obesity inherently promotes macrophage recruitment, MH obese individuals exhibit a distinct anti-inflammatory (M2) macrophage profile facilitated by adipocyte cytokine secretion that supports insulin sensitivity and control adipogenesis [46]. This delicate balance between pro- and anti-inflammatory macrophage polarization, influenced by environmental factors and gene regulation, ultimately dictates the extent of adipose tissue remodeling and its impact on metabolic health. In fact, our functional enrichment results of the DETs in the various groups and comparisons revealed a strong association with the processes and components of ECM remodeling and inflammation that emerged as the predominant findings in our comparative analysis.

Therefore, we next wanted to know if there were sex-specific differences even in the molecular signature of the processes that men and women with obesity share. Heparin-binding was the only shared component related to the ECM remodeling, but the transcripts that were up or downregulated were specific for each sex, with no common transcripts. Similarly, regarding inflammation, the analysis of females *vs.* males only revealed two common components. Analyzing the altered transcripts, we observed that the vWAT of obese females, independently of the metabolic status, presented higher inflammation and immune cell recruitment (*CCL15, CXCL10, C3, C5AR1, PRKCA, PRKD2*); ECM remodeling and fibrosis (*COL5A1, PCOLCE, FN1, VTN, EMILIN2, WNT5A*) and metabolic stress (*MDK, APLP1, JUP*). The vWAT of males, on the other side, presented lower inflammation (*HSPB6, BRCA1*), lower ECM remodeling (*PRELP, SFRP1*), and better vascularization and response to hypoxia (*VEGFB, ACVRL1, RRAS*). This observation corroborates the principle that effective neovascularization and requisite ECM remodeling mitigate hypoxia, thereby supporting adipose tissue homeostasis [47].

Our second goal was to analyze the transition from MH to MU obesity, which also revealed sex differences. First, the number of DETs between MH and MU (both for females and males) was tenfold lower than when we compared females *vs.* males, reinforcing our hypothesis that obesity should be treated as a different entity in women and men. Probably as a mechanism to prevent the ectopic accumulation of fat in females, the initial demand to store energy within the vWAT of MH patients provoked a significant deleterious response characterized by an increased immune activation. In males, the initial deposition of triglycerides in the vWAT does not cause the same pathologic response, suggesting that vWAT in males is better adapted to fat accumulation. In fact, MH males, in comparison to MU presented a reduction in proinflammatory pathways (including interferon-mediated pathways, as well as *IL-12* and *NOD1/2*) and an upregulation in anti-inflammatory processes, such as those mediated by *IL-10* and M2 alternative macrophage activation. Other stress-related pathways (renin-angiotensin system, *IGF/IGFBP*, *endothelin-1* or *NRF2*-mediated oxidative stress response) were also diminished in MH male patients.

In conclusion, our study has identified the molecular signatures of obese vWAT in males and females with differential metabolic profiles. According to our results, MH females presented the most deleterious profile, characterized by inflammation, fibrosis, and metabolic dysfunction, with MU males, MU females, and MH males following in this rank. Although the pathological processes that affect all of them are similar, there is a specific signature that characterizes each cohort, and this should be considered as criteria to establish an early treatment. However, several questions remain unanswered. For instance, it is yet to be determined what are the potential mechanisms that extend the expansion of adipose tissue of MH obese male patients, conferring them with a certain extent of protection. It is possible that a combination of epigenetic, genetic, transcriptomic and environmental factors lies in the base of the individual capacity to expand the adipose tissue, which impacts the biology of the adipocyte.

## Supplementary Information

Additional file 1.

Additional file 2.

Additional file 3.

Additional file 4.

Additional file 5.

Additional file 6.

Additional file 7.

Additional file 8.

Additional file 9.

Additional file 10.

Additional file 11.

## Data Availability

The dataset(s) supporting the conclusions of this article is(are) available in the CEU Repositorio Institucional repository, https://hdl.handle.net/10637/18643. "The dataset(s) supporting the conclusions of this article is(are) included within the article (and its additional file(s))."
