# Supplementary material for: Understanding the role of visceral fat in metabolically healthy versus unhealthy obesity: a sex-based analysis of the transcriptome
Source: Biol Sex Differ. 2025 Nov 6;16:92. doi: 10.1186/s13293-025-00777-6 (PMC12593901; doi:10.1186/s13293-025-00777-6)
Supplement: Supplementary file 1 — Additional file 1. [file 13293_2025_777_MOESM1_ESM.docx]

| Supplementary Table 1. Description of the cohorts included in the transcriptome analysis | | | | | | |
| --- | --- | --- | --- | --- | --- | --- |
|  | MH | MU | MH | | MU | |
|  | All (n=8) | All (n=8) | Male (n=4) | Female (n=4) | Male (n=4) | Female (n=4) |
| *Population Description* |  |  |  |  |  |  |
| Age (years) | 33.12 ± 3.1 | 42.00 ± 2.17 * | 37.00 ± 2.80 | 29.25 ± 5.15 | 43.00 ± 1.96 | 41.00 ± 4.18 |
| BMI (kg/m^2^) | 42.93 ± 0.80 | 44.50 ± 1.74 | 43.25 ± 1.18 | 42.60 ± 1.22 | 45.43 ± 1.75 | 43.55 ± 3.23 |
| *Glucose homeostasis* |  |  |  |  |  |  |
| Glucose (mg/dL) | 92.13 ± 4.46 | 108.5 ± 13.64 | 94.00 ± 8.50 | 90.25 ± 4.27 | 95.75 ± 3.33 | 121.25 ± 27.37 |
| Insulin (mIU/L) | 16.93 ± 1.79 | 28.33 ± 6.82 | 20.18 ± 1.01 | 12.61 ± 2.02 | 20.55 ± 3.36 | 36.10 ± 12.87 |
| HOMA-IR index | 3.85 ± 0.2 | 8.69 ± 3.44 | 4.70 ± 0.58 | 2.73 ± 0.30 | 4.90 ± 0.91 | 12.47 ± 6.70 |
| HOMA-B index | 244.10 ± 41.86 | 271.0 ± 75.59 | 278.0 ± 56.77 | 199.00 ± 63.54 | 229.75 ± 24.87 | 312.25 ± 157.84 |
| *Lipid profile* |  |  |  |  |  |  |
| Triglycerides (mg/dL) | 107.1 ±12.97 | 155.3 ± 27.09 | 97.25 ± 21.87 | 117.0 ± 15.54 | 137.75 ± 30.02 | 172.75 ± 48.16 |
| Total cholesterol (mg/dL) | 156.4 ± 11.71 | 165.5 ± 5.97 | 160.25 ± 4.25 | 152.5 ± 24.74 | 165.00 ± 11.14 | 166.0 ± 6.49 |
| HDL-cholesterol (mg/dL) | 45.00 ± 3.92 | 42.57 ± 2.33 | 50.00 ± 7.22 | 40.00 ± 1.68 | 41.25 ± 3.54 | 44.33 ± 3.18 |
| LDL-cholesterol (mg/dL) | 93.50 ± 10.16 | 91.00 ± 7.95 | 97.75 ± 5.36 | 89.25 ± 21.01 | 91.67 ± 11.05 | 90.50 ± 12.65 |
| VLDL-cholesterol (mg/dL) | 22.88 ± 3.57 | 32.89 ± 5.91 | 22.35 ± 7.03 | 23.40 ± 3.11 | 30.80 ± 7.14 | 34.35 ± 9.74 |
| *Kidney function* |  |  |  |  |  |  |
| Creatinine (mg/dL) | 0.87 ± 0.05 | 0.79 ± 0.05 | 0.98 ± 0.02 | 0.77 ± 0.05 ^+^ | 0.88 ± 0.03 | 0.71 ± 0.06 |
| Albumin (g/dL) | 4.59 ± 0.08 | 4.80 ± 0.11 | 4.67 ± 0.07 | 4.50 ± 0.14 | 4.87 ± 0.17 | 4.75 ± 0.14 |
| *Ions* |  |  |  |  |  |  |
| Na^+^ (mmol/L) | 141.1 ± 0.69 | 141.9 ± 0.93 | 140.5 ± 0.87 | 141.75 ± 1.11 | 142.00 ± 1.08 | 141.75 ± 1.70 |
| K^+^ (mmol/L) | 4.51 ± 0.09 | 4.46 ± 0.10 | 4.61 ± 0.12 | 4.41 ± 0.14 | 4.60 ± 0.15 | 4.32 ± 0.09 |
| Cl^-^ (mmol/L) | 104.1 ± 0.55 | 104.0 ± 0.89 | 104.25 ± 1.03 | 104.00 ± 0.58 | 103.00 ± 0.91 | 105.00 ± 1.47 |
| Ca^2+^ (mmol/L) | 9.47 ± 0.11 | 9.62 ± 0.12 | 9.52 ± 0.18 | 9.42 ± 0.14 | 9.70 ± 0.22 | 9.55 ± 0.11 |
| P^+^ (mmol/L) | 3.20 ± 0.26 | 3.30 ± 0.19 | 3.15 ± 0.40 | 3.25 ± 0.40 | 3.08 ± 0.31 | 3.52 ± 0.18 ^$^ |
| Mg^2+^ (mmol/L) | 2.04 ± 0.05 | 1.99 ± 0.07 | 2.02 ± 0.09 | 2.07 ± 0.06 | 2.04 ± 0.09 | 1.94 ± 0.11 |
| *Liver function* |  |  |  |  |  |  |
| GPT (U/L) | 19.00 ± 3.32 | 24.98 ± 4.13 | 21.75 ± 6.44 | 16.25 ± 2.25 | 33.50 ± 3.97 | 16.45 ± 3.92 |
| GGT (U/L) | 20.00 ± 2.29 | 37.00 ± 6.73 * | 24.25 ± 2.87 | 15.75 ± 2.06 | 34.75 ± 8.17 | 39.25 ± 11.90 |
| ALP (U/L) | 77.38 ± 4.12 | 66.13 ± 5.21 | 77.25 ± 6.30 | 77.50 ± 6.31 | 71.50 ± 8.84 | 60.75 ± 5.41 |
| LDH (U/L) | 169.40 ± 13.79 | 162.4 ± 10.54 | 174.33 ± 16.71 | 165.75 ± 22.71 | 182.25 ± 9.70 | 142.50 ± 12.71 |
| *Hormones* |  |  |  |  |  |  |
| Cortisol (µg/dL) | 7.92 ± 0.82 | 11.37 ± 1.61 | 8.17 ± 1.03 | 7.40 ± 1.80 | 11.35 ± 1.49 | 11.40 ± 3.70 |
| *Inflammation* |  |  |  |  |  |  |
| C-reactive Protein (mg/L) | 6.58 ± 0.79 | 3.77 ± 0.90 | 4.03 ± 1.82 | 5.75 ± 1.69 | 3.83 ± 1.04 | 3.70 ± 1.89 |
| Sedimentation velocity (mm/h) | 11.29 ± 2.88 | 21.38 ± 7.88 | 9.00 ± 2.20 | 14.33 ± 6.36 | 7.00 ± 1.08 | 35.75 ± 12.27 |
| *Coagulation* |  |  |  |  |  |  |
| INR | 1.01 ± 0.01 | 1.03 ± 0.02 | 1.03 ± 0.02 | 0.99 ± 0.02 | 1.03 ± 0.03 | 1.03 ± 0.02 |
| Prothrombin time (s) | 100.0 ± 3.02 | 95.46 ± 3.37 | 95.70 ± 3.96 | 104.32 ± 3.81 | 94.28 ± 5.77 | 96.65 ± 4.33 |
| Activated Partial Thromboplastin Time (s) | 28.54 ± 0.78 | 28.75 ± 0.77 | 28.98 ± 1.49 | 28.10 ± 0.73 | 29.40 ± 1.30 | 28.10 ± 0.91 |
| HOMA-IR (Glucose (mmol/l) x Insulin (mIU/L) / 22.5  HOMA-B (20 x Insulin (miU/L) / (Glucose (mmol/L) - 3.5  * p<0.05, **p<0.01 MH vs MU (all patients included)  + p<0.05 Female vs male  $ p<0.05 MU vs MH | | | | | | |
