# Supplementary material for: Understanding the role of visceral fat in metabolically healthy versus unhealthy obesity: a sex-based analysis of the transcriptome
Source: Biol Sex Differ. 2025 Nov 6;16:92. doi: 10.1186/s13293-025-00777-6 (PMC12593901; doi:10.1186/s13293-025-00777-6)
Supplement: Supplementary file 2 — Additional file 2. [file 13293_2025_777_MOESM2_ESM.docx]

| **Supplementary Table S2. Significant Ingenuity Canonical Pathways and their respective genes in the MH females vs. MH males.** | | | |
| --- | --- | --- | --- |
| Ingenuity Canonical Pathways | -log(p-value) | z-score | Genes |
| Cell surface interactions at the vascular wall | 1,09E01 | 5,014 | ATP1B2,CD99,COL1A1,COL1A2,FCER1G,FN1,GAS6,GRB7,IGHA1,IGHV3-33,IGKV1-16,IGKV1-5,IGKV1D-16,IGKV3-11,IGKV3D-20,IGKV4-1,IGLV1-40,IGLV2-18,IGLV2-23,IGLV5-45,ITGAM,JCHAIN,MIF,PIK3R2,PROCR,PTPN6,SDC4,TGFB1,THBD |
| Syndecan interactions | 8,34E00 | 2,530 | COL1A1,COL1A2,COL5A1,FGF2,FN1,ITGB4,PRKCA,SDC4,TGFB1,VTN |
| Complement cascade | 7,6E00 | 2,449 | C1QA,C5AR1,C7,CR1,IGHG2,IGHG4,IGHV3-33,IGKV1-16,IGKV1-5,IGKV1D-16,IGKV3-11,IGKV3D-20,IGKV4-1,IGLV1-40,IGLV2-18,IGLV2-23,IGLV5-45,SERPING1,VTN |
| Fc epsilon receptor (FCERI) signaling | 6,84E00 | 4,379 | CDC34,FCER1G,FOS,IGHV3-33,IGKV1-16,IGKV1-5,IGKV1D-16,IGKV3-11,IGKV3D-20,IGKV4-1,IGLV1-40,IGLV2-18,IGLV2-23,IGLV5-45,JUN,LCP2,NAD ,NFATC2,PIK3R2,PSMB8,PSMC5,PSME1,VAV1 |
| Binding and Uptake of Ligands by Scavenger Receptors | 6,48E00 | 4,123 | COL1A1,COL1A2,COLEC12,IGHA1,IGHV3-33,IGKV1-16,IGKV1-5,IGKV1D-16,IGKV3-11,IGKV3D-20,IGKV4-1,IGLV1-40,IGLV2-18,IGLV2-23,IGLV5-45,JCHAIN,STAB2 |
| Fcgamma receptor (FCGR) dependent phagocytosis | 6,22E00 | 3,900 | ABI2,HCK,IGHG2,IGHG4,IGHV3-33,IGKV1-16,IGKV1-5,IGKV1D-16,IGKV3-11,IGKV3D-20,IGKV4-1,IGLV1-40,IGLV2-18,IGLV2-23,IGLV5-45,NAD ,PIK3R2,VAV1,WIPF2 |
| Immunoregulatory interactions between a Lymphoid and a non-Lymphoid cell | 6,11E00 | 3,710 | CD300LG,CD99,CLEC2B,COL1A1,COL1A2,COLEC12,HCST,IGHV3-33,IGKV1-16,IGKV1-5,IGKV1D-16,IGKV3-11,IGKV3D-20,IGKV4-1,IGLV1-40,IGLV2-18,IGLV2-23,IGLV5-45,NECTIN2,NPDC1,PILRA |
| Interleukin-4 and Interleukin-13 signaling | 6,1E00 | 1,291 | ALOX5,CCL2,COL1A2,FGF2,FN1,FOS,FSCN1,IGHG4,ITGAM,JUNB,MAOA,MUC1,TGFB1,TIMP1,TP53 |
| Eukaryotic Translation Initiation | 5,4E00 | 2,840 | CP,EIF1AX,EIF4EBP1,RPL19,RPL27,RPL30,RPL36,RPS13,RPS15A,RPS16,RPS19,RPS2,RPS27,RPS4Y1,RPS5 |
| Neutrophil degranulation | 5,02E00 | 5,303 | ACTR1B,ADGRE5,ALOX5,APEH,APRT,B4GALT1,BST1,BST2,C5AR1,CD14,CNN2,CR1,CST3,FCER1G,FGL2,GMFG,GSTP1,IMPDH2,ITGAM,JUP,MIF,NHLRC3,PTPN6,PYCARD,QSOX1,RAB3D,S100A11,S100A8,SIGLEC14,SLPI,TRAPPC1,VAMP8 |
| Signaling by the B Cell Receptor (BCR) | 4,62E00 | 3,638 | BLNK,IGHV3-33,IGKV1-16,IGKV1-5,IGKV1D-16,IGKV3-11,IGKV3D-20,IGKV4-1,IGLV1-40,IGLV2-23,NAD ,NFATC2,PSMB8,PSMC5,PSME1,PTPN6,VAV1 |
| Eukaryotic Translation Elongation | 4,6E00 | 2,887 | RPL19,RPL27,RPL30,RPL36,RPS13,RPS15A,RPS16,RPS19,RPS2,RPS27,RPS4Y1,RPS5 |
| SRP-dependent cotranslational protein targeting to membrane | 4,56E00 | 3,051 | RPL19,RPL27,RPL30,RPL36,RPS13,RPS15A,RPS16,RPS19,RPS2,RPS27,RPS4Y1,RPS5,SSR4 |
| Eukaryotic Translation Termination | 4,55E00 | 2,887 | RPL19,RPL27,RPL30,RPL36,RPS13,RPS15A,RPS16,RPS19,RPS2,RPS27,RPS4Y1,RPS5 |
| Response of EIF2AK4 (GCN2) to amino acid deficiency | 4,3E00 | 2,887 | RPL19,RPL27,RPL30,RPL36,RPS13,RPS15A,RPS16,RPS19,RPS2,RPS27,RPS4Y1,RPS5 |
| B Cell Receptor Signaling | 4,27E00 | 1,667 | BLNK,CREB3L1,CSK,EGR1,IGHA1,IGHG2,IGHG4,IGHV1-18,IGHV3-33,IGHV3-38,IGHV3-72,IGKV1-16,IGKV1-37,IGKV1-5,IGKV1-6,IGKV1D-16,IGKV2-24,IGKV2-40,IGKV2D-24,IGKV3-11,IGKV3D-15,IGKV3D-20,IGKV3D-7,IGKV4-1,IGLJ2,IGLJ3,IGLV1-40,IGLV2-18,IGLV2-23,IGLV5-45,JCHAIN,JUN,NFATC2,PIK3R2,PTEN,PTPN6,VAV1 |
| Regulation of Insulin-like Growth Factor (IGF) transport and uptake by IGFBPs | 4,1E00 | 3,051 | CP,CST3,FN1,GAS6,IGFBP2,IGFBP4,IGFBP6,MSLN,PAPPA,QSOX1,TF,TIMP1,TMEM132A |
| Extracellular matrix organization | 4,06E00 | 2,309 | BGN,COL1A1,COL1A2,COL5A1,COL6A6,DMD,FGF2,FN1,ITGB4,PDGFB,TGFB1,VTN |
| Major pathway of rRNA processing in the nucleolus and cytosol | 3,99E00 | 3,500 | DDX49,FBL,NOB1,RPL19,RPL27,RPL30,RPL36,RPS13,RPS15A,RPS16,RPS19,RPS2,RPS27,RPS4Y1,RPS5,SNORD3A |
| NAD Signaling Pathway | 3,9E00 | 1,732 | BST1,ERN1,FGF2,H1-0,H1-10,H2BC26,NAD ,PDGFB,PIK3R2,POLR2G,POLR2I,POLR2L,SLC36A4,TGFB1,TP53 |
| PI3K Signaling in B Lymphocytes | 3,88E00 | 2,646 | BLNK,FOS,IGHA1,IGHG2,IGHG4,IGHV1-18,IGHV3-33,IGHV3-38,IGHV3-72,IGKV1-16,IGKV1-37,IGKV1-5,IGKV1-6,IGKV1D-16,IGKV2-24,IGKV2-40,IGKV2D-24,IGKV3-11,IGKV3D-15,IGKV3D-20,IGKV3D-7,IGKV4-1,IGLJ2,IGLJ3,IGLV1-40,IGLV2-18,IGLV2-23,IGLV5-45,JUN,NFATC2,PIK3R2,PLEKHA4,PTEN,VAV1 |
| Nonsense-Mediated Decay (NMD) | 3,74E00 | 2,887 | RPL19,RPL27,RPL30,RPL36,RPS13,RPS15A,RPS16,RPS19,RPS2,RPS27,RPS4Y1,RPS5 |
| Neutrophil Extracellular Trap Signaling Pathway | 3,7E00 | 3,138 | ATP5F1D,C1QA,CD14,COL1A1,COL1A2,COL5A1,COL6A6,CYC1,HCK,IGHA1,IGHG2,IGHG4,ITGAM,JCHAIN,KCNN3,NDUFA11,NDUFA13,NDUFA7,NDUFB11,NDUFB3,PIK3R2,PLAAT4,PRKCA,PYCARD,SLC25A6,TIMM9 |
| GPVI-mediated activation cascade | 3,68E00 | 2,646 | COL1A1,COL1A2,FCER1G,LCP2,PIK3R2,PTPN6,VAV1 |
| Granzyme A Signaling | 3,57E00 | -3,000 | FN1,H1-0,H1-10,NDUFA1,NDUFA11,NDUFA13,NDUFA7,NDUFB11,NDUFB3 |
| PCP (Planar Cell Polarity) Pathway | 3,51E00 | 1,414 | FZD7,JUN,JUNB,JUND,LGR4,PRICKLE1,SMO,WNT5A |
| IL-17A Signaling in Fibroblasts | 3,31E00 | 2,333 | CCL2,COL1A1,COL1A2,FN1,FOS,JUN,PRKCA,TGFB1,THY1 |
| WNT/β-catenin Signaling | 3,27E00 | 0,277 | BCL9,CDH3,DKK1,FZD7,JUN,PPP2R1B,RARA,RARG,RUVBL2,SFRP4,SMO,TGFB1,TP53,WNT5A |
| Macrophage Alternative Activation Signaling Pathway | 3,21E00 | 2,840 | CREB3L1,CSF1R,DUSP1,EIF4EBP1,FCER1G,FOS,IGHG2,IGHG4,IL10RA,JCHAIN,JUN,KLF4,MLST8,PIK3R2,TGFB1 |
| Complement System | 3,15E00 | 0,000 | C1QA,C5AR1,C7,CR1,ITGAM,SERPING1 |
| Hereditary Breast Cancer Signaling | 3,12E00 | 0,000 | FANCL,FGF2,H2AX,NELFB,PDGFB,PIK3R2,POLR2G,POLR2I,POLR2L,PTEN,TGFB1,TP53 |
| Processing of Capped Intron-Containing Pre-mRNA | 3,07E00 | 2,982 | LSM2,LSM5,LSM7,LUC7L3,POLR2G,POLR2I,POLR2L,PPIL1,RNPC3,RNU12,RNU4ATAC,RNU5B-1,SF3A2,SNRPB,SNRPC,SNRPD1,SNRPD2,SRSF11,UBL5 |
| Autophagy | 3 | 0,775 | CREB3L1,DRAM1,ERN1,FGF2,FOS,FUNDC1,JUN,MLST8,NGFR,PDGFB,PIK3R2,PPP2R1B,PTEN,TGFB1,TP53,VAMP8 |
| ESR-mediated signaling | 2,97E00 | 2,714 | CARM1,FOS,GREB1,H2AX,H2BC26,JUN,JUND,POLR2G,POLR2I,POLR2L,TNRC6C |
| TP53 Regulates Transcription of DNA Repair Genes | 2,97E00 | 2,828 | FOS,JUN,NELFB,POLR2G,POLR2I,POLR2L,SUPT4H1,TP53 |
| CSDE1 Signaling Pathway | 2,96E00 | 1,134 | CCL2,EDC4,FN1,FOS,PTEN,SDC4,TGFB1 |
| Post-translational protein phosphorylation | 2,91E00 | 2,530 | CP,CST3,FN1,GAS6,IGFBP4,MSLN,QSOX1,TF,TIMP1,TMEM132A |
| Electron transport, ATP synthesis, and heat production by uncoupling proteins | 2,91E00 | 2,887 | ATP5F1D,ATP5ME,CYC1,ECSIT,NAD ,NDUFA1,NDUFA11,NDUFA13,NDUFA7,NDUFB11,NDUFB3,SLC25A27 |
| EIF2 Signaling | 2,9E00 | 2,449 | EIF1AX,EIF1AY,PIK3R2,PPP1R15A,RPL19,RPL27,RPL30,RPL36,RPS13,RPS15A,RPS16,RPS19,RPS2,RPS27,RPS4Y1,RPS5 |
| PIP3 activates AKT signaling | 2,88E00 | 1,732 | CD28,FGF2,FLT3LG,IER3,MLST8,NR4A1,PDGFB,PIK3R2,PIP4K2C,PPP2R1B,PTEN,VAV1 |
| RET signaling | 2,86E00 | 2,449 | DOK1,DOK4,GRB7,PIK3R2,PRKCA,SHC3 |
| Oxidative Stress Induced Senescence | 2,84E00 | 2,121 | CBX6,CDKN2B,FOS,H2AX,H2BC26,JUN,RING1,TNRC6C,TP53 |
| Cell junction organization | 2,81E00 | 2,333 | ARHGEF6,CADM3,CDH11,CDH3,CLDN15,FLNC,ITGB4,JUP,NECTIN2 |
| Selenoamino acid metabolism | 2,76E00 | 2,887 | RPL19,RPL27,RPL30,RPL36,RPS13,RPS15A,RPS16,RPS19,RPS2,RPS27,RPS4Y1,RPS5 |
| PTEN Regulation | 2,67E00 | 1,000 | CBX6,EGR1,JUN,MLST8,NAD ,PSMB8,PSMC5,PSME1,PTEN,RING1,TNRC6C,TP53 |
| RAR Activation | 2,62E00 | 1,633 | ADCY9,ADH1B,CARM1,CDKN2B,COL1A1,COL1A2,CRABP2,CREB3L1,CSK,DKK1,DUSP1,FOS,JUN,KLF2,PDE3B,PIK3R2,PSMC5,PTEN,RARA,RARG,SORBS3,TGFB1,TGM2,ZBTB16 |
| Assembly of collagen fibrils and other multimeric structures | 2,58E00 | 2,646 | COL14A1,COL1A1,COL1A2,COL5A1,COL6A6,ITGB4,PCOLCE |
| Signaling by SCF-KIT | 2,56E00 | 1,633 | FER,GRB7,PIK3R2,PRKCA,PTPN6,VAV1 |
| Oxidative Phosphorylation | 2,55E00 | 3,162 | ATP5F1D,ATP5ME,CYC1,NAD ,NDUFA1,NDUFA11,NDUFA13,NDUFA7,NDUFB11,NDUFB3 |
| Sirtuin Signaling Pathway | 2,54E00 | -0,535 | ATP5F1D,CYC1,H1-0,H1-10,HSF1,JUN,NAD ,NDUFA1,NDUFA11,NDUFA13,NDUFA7,NDUFB11,NDUFB3,NQO1,SF3A2,SLC25A6,TIMM9,TP53,TRIM28 |
| Response to elevated platelet cytosolic Ca2+ | 2,53E00 | 3,317 | FN1,GAS6,LGALS3BP,MMRN1,PDGFB,PRKCA,QSOX1,SERPING1,TF,TGFB1,TIMP1 |
| Phospholipase C Signaling | 2,53E00 | 1,667 | ADCY9,ARHGEF6,BLNK,CREB3L1,FCER1G,GNG7,GPLD1,IGHA1,IGHG2,IGHG4,IGHV1-18,IGHV3-33,IGHV3-38,IGHV3-72,IGKV1-16,IGKV1-37,IGKV1-5,IGKV1-6,IGKV1D-16,IGKV2-24,IGKV2-40,IGKV2D-24,IGKV3-11,IGKV3D-15,IGKV3D-20,IGKV3D-7,IGKV4-1,IGLJ2,IGLJ3,IGLV1-40,IGLV2-18,IGLV2-23,IGLV5-45,ITGAM,ITGB4,JCHAIN,LCP2,NFATC2,PRKCA,TGM2,TRAJ23,TRAJ32,TRAJ35,TRAJ37,TRAJ39,TRBJ2-4,TRBV20-1,TRDC,TRDJ2 |
| WNT/Ca+ pathway | 2,44E00 | 2,449 | CREB3L1,FZD7,NFATC2,PRKCA,ROR1,SMO,WNT5A |
| Pre-NOTCH Expression and Processing | 2,41E00 | 1,890 | B4GALT1,H2AX,H2BC26,JUN,NAD ,RFNG,TNRC6C,TP53 |
| mTOR Signaling | 2,31E00 | 1,633 | EIF4EBP1,GPLD1,MLST8,PIK3R2,PPP2R1B,PRKCA,RPS13,RPS15A,RPS16,RPS19,RPS2,RPS27,RPS4Y1,RPS5 |
| Insertion of tail-anchored proteins into the endoplasmic reticulum membrane | 2,27E00 | 2,000 | CAMLG,GET3,PRNP,VAMP2 |
| Collagen biosynthesis and modifying enzymes | 2,27E00 | 1,890 | COL14A1,COL1A1,COL1A2,COL5A1,COL6A6,PCOLCE,PLOD2 |
| Hypoxia Signaling in the Cardiovascular System | 2,2E00 | 0,000 | CDC34,CREB3L1,JUN,NQO1,PTEN,TP53,UBE2M |
| IL-13 Signaling Pathway | 2,19E00 | 1,667 | ANO1,DEFB128,DUSP1,HCK,JUN,MAOA,PIK3R2,TGFB1,TP53 |
| Senescence Pathway | 2,13E00 | 0,243 | CAPN10,CDC25B,CDKN2B,DHCR24,DMTF1,EIF4EBP1,ELF4,JUN,NFATC2,PCGF2,PIK3R2,PPP2R1B,PTEN,RING1,TGFB1,TP53,YPEL3 |
| Role of NFAT in Regulation of the Immune Response | 2,13E00 | 2,828 | BLNK,CD28,FCER1G,FOS,GNG7,IGHA1,IGHG2,IGHG4,IGHV1-18,IGHV3-33,IGHV3-38,IGHV3-72,IGKV1-16,IGKV1-37,IGKV1-5,IGKV1-6,IGKV1D-16,IGKV2-24,IGKV2-40,IGKV2D-24,IGKV3-11,IGKV3D-15,IGKV3D-20,IGKV3D-7,IGKV4-1,IGLJ2,IGLJ3,IGLV1-40,IGLV2-18,IGLV2-23,IGLV5-45,JUN,LCP2,NFATC2,PIK3R2,TRAJ23,TRAJ32,TRAJ35,TRAJ37,TRAJ39,TRBJ2-4,TRBV20-1,TRDC,TRDJ2 |
| DNA Damage/Telomere Stress Induced Senescence | 2,13E00 | 2,449 | ACD,H1-0,H2AX,H2BC26,HMGA1,TP53 |
| Wound Healing Signaling Pathway | 2,12E00 | 2,840 | COL1A1,COL1A2,COL5A1,COL6A6,FGF2,FN1,FOS,ITGB4,JUN,NGFR,PDGFB,PRKCA,SHC3,TGFB1,TRADD |
| TCF dependent signaling in response to WNT | 2,11E00 | 2,496 | BCL9,DKK1,FRAT2,H2AX,H2BC26,LGR4,NAD ,PPP2R1B,PSMB8,PSMC5,PSME1,PYGO2,WNT5A |
| Aryl Hydrocarbon Receptor Signaling | 2,09E00 | -1,134 | CYP3A4,FOS,GSTM5,GSTP1,JUN,NQO1,RARA,RARG,TGFB1,TGM2,TP53 |
| Acute Phase Response Signaling | 2,09E00 | 1,414 | C1QA,CP,CRABP2,ECSIT,FN1,FOS,JUN,NGFR,PIK3R2,SERPING1,TF,TRADD |
| Collagen chain trimerization | 2,08E00 | 2,236 | COL14A1,COL1A1,COL1A2,COL5A1,COL6A6 |
| Phagosome Formation | 2,07E00 | 4,131 | ADGRE5,ADRA2C,C5AR1,CD14,COLEC12,CR1,CYSLTR2,FCER1G,FN1,FZD7,GPLD1,GPRC5B,HCK,IGHA1,IGHG2,IGHG4,ITGAM,ITGB4,JCHAIN,LGR4,LHCGR,MRC2,NPY1R,PIK3R2,PIP4K2C,PLAAT4,PRKCA,PTGER2,S1PR2,SMO,VAV1,VTN |
| Elastic fibre formation | 2,03E00 | 2,236 | EMILIN2,FBLN1,FN1,TGFB1,VTN |
| Estrogen Receptor Signaling | 2,03E00 | 2,840 | ADCY9,ATP5F1D,CARM1,CREB3L1,CYC1,EIF4EBP1,FOS,GNG7,JUN,MDK,MMP24,NDUFA11,NDUFA13,NDUFA7,NDUFB11,NDUFB3,PIK3R2,PRKCA,PTEN,SHC3,TP53 |
| IL-15 Production | 2,03E00 | 2,333 | AXL,CSF1R,CSK,EPHB2,FER,FLT3LG,HCK,PTK7,ROR1 |
| IL-12 Signaling and Production in Macrophages | 1,98E00 | -1,069 | COL1A1,COL1A2,FOS,IGHG2,IGHG4,IL10RA,ITGAM,JCHAIN,JUN,NFATC2,PIK3R2,PRKCA,S100A8,TGFB1 |
| Collagen degradation | 1,96E00 | 2,449 | COL14A1,COL1A1,COL1A2,COL5A1,COL6A6,CTSK |
| Signaling by PDGF | 1,96E00 | 2,449 | COL5A1,COL6A6,GRB7,PDGFB,PIK3R2,PLAT |
| MIF Regulation of Innate Immunity | 1,96E00 | 1,342 | CD14,FOS,JUN,MIF,TP53 |
| Toll-like Receptor Cascades | 1,94E00 | 2,000 | CD14,DNM1,ITGAM,LY86 |
| Integrin cell surface interactions | 1,92E00 | 2,646 | COL1A1,COL1A2,COL5A1,COL6A6,FN1,ITGAM,VTN |
| Costimulation by the CD28 family | 1,92E00 | 1,890 | CD28,CSK,MLST8,PIK3R2,PPP2R1B,PTPN6,VAV1 |
| Signaling by VEGF | 1,88E00 | 2,333 | ABI2,AXL,JUP,MLST8,NAD ,PIK3R2,PRKCA,SHB,VAV1 |
| Renin-Angiotensin Signaling | 1,88E00 | 2,646 | ADCY9,CCL2,FOS,JUN,PIK3R2,PRKCA,PTGER2,PTPN6,SHC3 |
| Mitochondrial Dysfunction | 1,87E00 | -2,524 | ATP1B2,ATP5F1D,ATP5ME,CAPN10,CREB3L1,CYC1,GPX3,GSTP1,HSF1,MAOA,NAD ,NDUFA1,NDUFA11,NDUFA13,NDUFA7,NDUFB11,NDUFB3,PIK3R2,TP53 |
| Communication between Innate and Adaptive Immune Cells | 1,87E00 | 3,900 | CD28,CXCL10,FCER1G,IGHA1,IGHG2,IGHG4,IGHV1-18,IGHV3-33,IGHV3-38,IGHV3-72,IGKV1-16,IGKV1-37,IGKV1-5,IGKV1-6,IGKV1D-16,IGKV2-24,IGKV2-40,IGKV2D-24,IGKV3-11,IGKV3D-15,IGKV3D-20,IGKV3D-7,IGKV4-1,IGLJ2,IGLJ3,IGLV1-40,IGLV2-18,IGLV2-23,IGLV5-45,JCHAIN,TRAJ23,TRAJ32,TRAJ35,TRAJ37,TRAJ39,TRBJ2-4,TRBV20-1,TRDC,TRDJ2 |
| GP6 Signaling Pathway | 1,84E00 | 2,828 | COL1A1,COL1A2,COL5A1,COL6A6,FCER1G,LCP2,PIK3R2,PRKCA,VAV1 |
| ILK Signaling | 1,82E00 | 2,111 | ARHGEF6,CREB3L1,FLNC,FN1,FOS,ITGB4,JUN,MUC1,PIK3R2,PPP2R1B,PTEN,TGFB1I1 |
| RNA polymerase II transcribes snRNA genes | 1,82E00 | 2,646 | POLR2G,POLR2I,POLR2L,RNU12,RNU4ATAC,RNU5B-1,SUPT4H1 |
| FAK Signaling | 1,77E00 | 3,395 | ADGRE5,ADRA2C,ARHGEF6,C5AR1,CAPN10,COL1A1,COL1A2,CSK,CYSLTR2,EFNA5,EFNB3,ELF4,FCER1G,FOS,FZD7,GHR,GPRC5B,IL10RA,IL17RD,ITGAM,ITGB4,JUN,LGR4,LHCGR,NPY1R,PIK3R2,PTEN,PTGER2,S1PR2,SHB,SMO,TGFB1,TP53,TRAJ23,TRAJ32,TRAJ35,TRAJ37,TRAJ39,TRBJ2-4,TRBV20-1,TRDC,TRDJ2 |
| STAT3 Pathway | 1,77E00 | -0,378 | FGF2,GHR,IL10RA,IL17RD,NDUFA13,NGFR,PDGFB,PTPN6,TGFB1 |
| Microautophagy Signaling Pathway | 1,73E00 | 2,449 | CHMP6,COL1A1,COL1A2,COL5A1,COL6A6,PSMB8,PSMC5,PSME1,TP53,VAMP8 |
| Corticotropin Releasing Hormone Signaling | 1,72E00 | 2,449 | ADCY9,CREB3L1,FOS,GLI3,JUN,JUND,NR4A1,NT5C,PRKCA,SMO |
| Ceramide Signaling | 1,7E00 | 0,816 | FOS,JUN,NGFR,PIK3R2,PPP2R1B,S1PR2,SMPD3 |
| Beta-catenin independent WNT signaling | 1,7E00 | 2,121 | AP2S1,FZD7,GNG7,PRICKLE1,PRKCA,ROR1,TNRC6C,WNT5A |
| NRF2-mediated Oxidative Stress Response | 1,69E00 | 1,633 | CDC34,CYP2S1,CYP3A4,DNAJB1,FOS,GSTM5,GSTP1,JUN,JUNB,JUND,NQO1,PIK3R2,PRKCA |
| MAPK6/MAPK4 signaling | 1,68E00 | 1,000 | CDC42EP5,DNAJB1,JUN,PSMB8,PSMC5,PSME1,TNRC6C |
| Growth Hormone Signaling | 1,68E00 | 0,000 | CSH1/CSH2,FOS,GHR,PIK3R2,PRKCA,PTPN6 |
| C-type lectin receptors (CLRs) | 1,67E00 | 2,828 | CDC34,FCER1G,MUC1,NAD ,NFATC2,PSMB8,PSMC5,PSME1,PYCARD,UBE2M |
| Protein Kinase A Signaling | 1,66E00 | 0,728 | ADCY9,CDC25B,CREB3L1,DUSP1,FLNC,GLI3,GNG7,H1-0,H1-10,NFATC2,NGFR,PDE3B,PRKCA,PTEN,PTPN23,PTPN3,PTPN6,PYGM,SMO,TGFB1 |
| Interleukin-3, Interleukin-5 and GM-CSF signaling | 1,62E00 | 2,236 | BLNK,HCK,PIK3R2,PTPN6,VAV1 |
| O-linked glycosylation | 1,6E00 | 1,414 | ADAMTS18,ADAMTS5,ADAMTS7,ADAMTSL5,B3GNT9,B3GNTL1,MUC1,ST3GAL2 |
| RHO GTPase cycle | 1,6E00 | 2,837 | ABI2,ABR,ARHGAP23,ARHGAP24,ARHGAP42,ARHGDIG,ARHGEF6,BASP1,CDC42EP5,CSK,GRB7,JUP,PIK3R2,PLEKHG2,RHPN2,STARD8,SYDE1,TAGAP,VAV1,WDR6,WIPF2 |
| Glycosaminoglycan metabolism | 1,59E00 | 2,646 | B4GALT1,BGN,CHPF,LYVE1,SDC4,ST3GAL2,STAB2 |
| Fcγ Receptor-mediated Phagocytosis in Macrophages and Monocytes | 1,59E00 | 1,134 | GPLD1,HCK,LCP2,PIK3R2,PRKCA,PTEN,VAV1 |
| NGF-stimulated transcription | 1,53E00 | 2,000 | EGR1,FOS,JUNB,JUND |
| RAF/MAP kinase cascade | 1,52E00 | 2,673 | CSK,DUSP1,FGF2,FLT3LG,FN1,IL17RD,PDGFB,PIK3R2,PPP2R1B,PSMB8,PSMC5,PSME1,PTPN3,SHC3 |
| ID1 Signaling Pathway | 1,48E00 | 1,508 | EGR1,FGF2,FN1,HCK,NGFR,PIK3R2,PTEN,PTK7,TGFB1,TGM2,TP53 |
| Role of JAK2 in Hormone-like Cytokine Signaling | 1,48E00 | -0,447 | BIRC3,CYP3A4,FOS,GHR,PTPN6 |
| Eicosanoid Signaling | 1,44E00 | 3,873 | ADCY9,ALOX5,CREB3L1,CYSLTR2,EGR1,GNG7,KLF4,NAD ,PIK3R2,PLAAT4,PRKCA,PTGDS,PTGER2,PTGIS,PTGS1 |
| G alpha (12/13) signalling events | 1,44E00 | 0,816 | ABR,ARHGEF35,ARHGEF6,GNG7,PLEKHG2,VAV1 |
| Erythropoietin Signaling Pathway | 1,41E00 | 1,000 | BIRC3,FOS,HBA1/HBA2,JUN,PIK3R2,PRKCA,PTPN6,SHC3,TGFB1,TP53 |
| Regulation of the Epithelial Mesenchymal Transition in Development Pathway | 1,4E00 | 2,236 | BCL9,FZD7,GLI3,PYGO2,SMO,WNT5A |
| LPS/IL-1 Mediated Inhibition of RXR Function | 1,4E00 | 2,236 | CD14,CHST15,CYP2S1,CYP3A4,ECSIT,FMO3,GSTM5,GSTP1,JUN,MAOA,NGFR,RARA,SLC27A1 |
| Atherosclerosis Signaling | 1,39E00 | 2,828 | ALOX5,CCL2,COL1A1,COL1A2,PDGFB,PLAAT4,S100A8,TGFB1 |
| Hedgehog 'on' state | 1,38E00 | 2,449 | DZIP1,GLI3,PSMB8,PSMC5,PSME1,SMO |
| TCR signaling | 1,38E00 | 2,121 | CDC34,CSK,LCP2,PIK3R2,PSMB8,PSMC5,PSME1,PTEN |
| Necroptosis Signaling Pathway | 1,37E00 | 2,333 | AXL,BIRC3,CAPN10,NGFR,PYCARD,SLC25A6,TIMM9,TP53,TRADD |
| Synthesis of Prostaglandins (PG) and Thromboxanes (TX) | 1,37E00 | 2,000 | NAD ,PTGDS,PTGIS,PTGS1 |
| Interleukin-10 signaling | 1,34E00 | 2,000 | CCL2,CXCL10,IL10RA,TIMP1 |
| HIF1α Signaling | 1,32E00 | 0,905 | EIF4EBP1,FGF2,JUN,MMP24,PDGFB,PIK3R2,PRKCA,SAT2,TF,TGFB1,TP53 |
