# Supplementary material for: Understanding the role of visceral fat in metabolically healthy versus unhealthy obesity: a sex-based analysis of the transcriptome
Source: Biol Sex Differ. 2025 Nov 6;16:92. doi: 10.1186/s13293-025-00777-6 (PMC12593901; doi:10.1186/s13293-025-00777-6)
Supplement: Supplementary file 3 — Additional file 3. [file 13293_2025_777_MOESM3_ESM.docx]

| **Supplementary Table S3. Significant Ingenuity Canonical Pathways and their respective genes in the MU females vs. MU males.** | | | |
| --- | --- | --- | --- |
| Ingenuity Canonical Pathways | -log(p-value) | z-score | Genes |
| Neddylation | 2,16E00 | 2.333 | BRCA1,BTBD6,DDA1,FBXO27,FBXO9,FBXW5,PSMB10,PSMB5,SPSB3 |
| Metabolism of polyamines | 2 | 1.000 | PSMB10,PSMB5,SAT1,SRM |
| WNT/β-catenin Signaling | 1,99E00 | -1.633 | CSNK1A1,CSNK1E,GNAO1,RARA,SFRP1,SFRP4,TLE1 |
| Sirtuin Signaling Pathway | 1,85E00 | -1.890 | FOXO3,HSF1,MLYCD,NDUFA1,NDUFA7,NDUFB1,NDUFB11,TIMM9,TOMM34 |
| Granzyme A Signaling | 1,83E00 | -2.000 | NDUFA1,NDUFA7,NDUFB1,NDUFB11 |
| TCF dependent signaling in response to WNT | 1,74E00 | 1.134 | CSNK1A1,CSNK1E,PSMB10,PSMB5,PYGO2,SFRP1,TLE1 |
| ID1 Signaling Pathway | 1,64E00 | 1.890 | ACVRL1,BHLHE40,FOXO3,NGFR,PTEN,RRAS,VEGFB |
| Degradation of beta-catenin by the destruction complex | 1,46E00 | 1.000 | CSNK1A1,PSMB10,PSMB5,TLE1 |
| Phase I - Functionalization of compounds | 1,38E00 | 1.000 | AOC2,CYP3A5,FMO3,MAOB |
| Transcriptional regulation by RUNX3 | 1,38E00 | 1.000 | FOXO3,PSMB10,PSMB5,ZFHX3 |
| PTEN Regulation | 1,31E00 | 1.000 | CBX2,PSMB10,PSMB5,PTEN,RRAGD |
| Necroptosis Signaling Pathway | 1,31E00 | 1.342 | NGFR,RNF31,SLC25A3,TIMM9,TOMM34 |
