# Supplementary material for: Understanding the role of visceral fat in metabolically healthy versus unhealthy obesity: a sex-based analysis of the transcriptome
Source: Biol Sex Differ. 2025 Nov 6;16:92. doi: 10.1186/s13293-025-00777-6 (PMC12593901; doi:10.1186/s13293-025-00777-6)
Supplement: Supplementary file 4 — Additional file 4. [file 13293_2025_777_MOESM4_ESM.docx]

| **Supplementary Table S4. Significant Ingenuity Canonical Pathways and their respective genes in the MH females vs. MU females.** | | | |
| --- | --- | --- | --- |
| Ingenuity Canonical Pathways | -log(p-value) | z-score | Genes |
| Binding and Uptake of Ligands by Scavenger Receptors | 5,42E00 | 2,236 | COL1A1,IGKV3D-20,IGKV4-1,IGLV2-18,STAB2 |
| Cell surface interactions at the vascular wall | 4,9E00 | 1,633 | ANGPT1,COL1A1,FN1,IGKV3D-20,IGKV4-1,IGLV2-18 |
| Communication between Innate and Adaptive Immune Cells | 3,57E00 | 2,449 | IGKV2-40,IGKV3D-15,IGKV3D-20,IGKV3D-7,IGKV4-1,IGLV2-18 |
| Immunoregulatory interactions between a Lymphoid and a non-Lymphoid cell | 2,87E00 | 2,000 | COL1A1,IGKV3D-20,IGKV4-1,IGLV2-18 |
| Fc epsilon receptor (FCERI) signaling | 2,77E00 | 2,000 | IGKV3D-20,IGKV4-1,IGLV2-18,JUN |
| RAR Activation | 1,52E00 | -1,000 | CDKN2B,COL1A1,JUN,RBP1 |
| Phagosome Formation | 1,38E00 | -1,342 | FN1,GPLD1,LHCGR,PLA2G4A,PTGER3 |
