# Supplementary material for: Understanding the role of visceral fat in metabolically healthy versus unhealthy obesity: a sex-based analysis of the transcriptome
Source: Biol Sex Differ. 2025 Nov 6;16:92. doi: 10.1186/s13293-025-00777-6 (PMC12593901; doi:10.1186/s13293-025-00777-6)
Supplement: Supplementary file 5 — Additional file 5. [file 13293_2025_777_MOESM5_ESM.docx]

| **Supplementary Table S5. Significant Ingenuity Canonical Pathways and their respective genes in the MH males vs. MU males.** | | | |
| --- | --- | --- | --- |
| Ingenuity Canonical Pathways | -log(p-value) | z-score | Molecules |
| Role of PKR in Interferon Induction and Antiviral Response | 4,65E00 | -1,342 | ATF3,FOS,HSPA2,JUN,NLRP1,PDGFD |
| NGF-stimulated transcription | 4,63E00 | -2,000 | EGR1,FOS,JUNB,SGK1 |
| ABRA Signaling Pathway | 3,33E00 | -2,000 | EGR1,FOS,HAND2,JUNB |
| Interleukin-4 and Interleukin-13 signaling | 2,89E00 | -1,000 | CCL2,FOS,JUNB,TIMP1 |
| Regulation of the Epithelial Mesenchymal Transition by Growth Factors Pathway | 2,88E00 | -2,000 | EGR1,FGF9,FOS,JUN,PDGFD |
| Renin-Angiotensin Signaling | 2,75E00 | -2,000 | CCL2,FOS,JUN,PRKCI |
| Regulation of Insulin-like Growth Factor (IGF) transport and uptake by IGFBPs | 2,7E00 | -1,000 | BMP4,FBN1,PAPPA,TIMP1 |
| IL-12 Signaling and Production in Macrophages | 2,6E00 | -1,342 | FOS,JUN,NFATC2,PRKCI,THBS1 |
| Apelin Endothelial Signaling Pathway | 2,53E00 | -2,000 | CCL2,FOS,JUN,PRKCI |
| PI3K Signaling in B Lymphocytes | 2,49E00 | -2,236 | ATF3,FOS,JUN,NFATC2,PRKCI |
| IL-10 Signaling | 2,45E00 | 1,000 | BHLHE40,DUSP1,FOS,JUN |
| Macrophage Alternative Activation Signaling Pathway | 2,19E00 | 1,000 | DUSP1,FOS,JUN,THBS1 |
| NOD1/2 Signaling Pathway | 2,17E00 | -2,000 | CCL2,FOS,HSPA2,JUN |
| Senescence Pathway | 2,07E00 | -1,000 | ATF3,BHLHE40,CAPN6,JUN,NFATC2 |
| Endothelin-1 Signaling | 2,06E00 | -1,000 | EDN1,FOS,JUN,PRKCI |
| GNRH Signaling | 2,06E00 | -2,000 | EGR1,FOS,JUN,PRKCI |
| NRF2-mediated Oxidative Stress Response | 1,89E00 | -1,000 | FOS,JUN,JUNB,PRKCI |
