# Supplementary material for: Understanding the role of visceral fat in metabolically healthy versus unhealthy obesity: a sex-based analysis of the transcriptome
Source: Biol Sex Differ. 2025 Nov 6;16:92. doi: 10.1186/s13293-025-00777-6 (PMC12593901; doi:10.1186/s13293-025-00777-6)
Supplement: Supplementary file 6 — Additional file 6. [file 13293_2025_777_MOESM6_ESM.docx]

| **Supplementary table S6. Functional enrichment analysis based on the related extracellular component of the differential transcript expression of the MH female vs. MH male** | | | | | |
| --- | --- | --- | --- | --- | --- |
| **Database** | **Related extracellular component** | **No. of genes** | **Fold enrichment** | ***p*-value** | **Genes** |
| Uniprot | Plasma membrane | 229 | 1.265387 | 2.28E-05 | P00325; P14209; Q6IEY1; Q8WWZ7; B2RN74; Q13606; P22888; Q9BXB1; Q9BZV2; Q8NGY9; Q6UXG8; Q8NGA8; P00450; Q13642; Q8NG94; P25929; Q8NH05; Q8NGJ6; P59542; A6NCV1; Q8TCB6; P10912; Q8IVW1; P59538; Q0ZGT2; Q6UXG3; P11532; Q8NGL9; Q9UGF6; Q05516; Q8NG97; O60669; Q9HC96; Q3KNW5; Q9NX77; Q9UIQ6; Q6ZWE6; O95876; A6NL99; Q6U736; Q6YBV0; A6NL26; Q8NGL3; P48960; P43116; Q15628; A0A087WSX0; Q92529; Q8TF74; O14735; P29323; P36269; Q14451; O95716; Q86XT9; Q15768; Q96CP6; O75074; Q8NFZ4; P60510; P04216; Q5XXA6; O00499; O60262; Q9NZH0; Q9UKJ1; Q15464; Q9BRK4; O95528; P01024; C9J798; O43374; Q8WWQ8; Q9BSA4; P15291; Q10588; Q96P69; Q9NQX5; P26045; Q6PCB7; P32418; Q3MIR4; Q86YJ7; Q13651; Q9BSK0; Q9H5I5; Q9NNW5; P51693; Q0VAQ4; P40617; Q92478; P14923; Q10589; Q9BV40; P15498; P53680; Q9Y4D2; Q05193; O43736; O95136; P63027; Q04941; P17252; Q96DZ5; P04156; Q99835; P08631; Q9BZL6; Q9UNN8; P13639; P30530; Q8WV28; Q8NFM7; Q01973; Q9NY59; Q5T9L3; Q96FZ7; P52803; Q8IVF2; Q9UGK3; Q15942; Q9NQX7; Q9Y5Y7; Q96NT5; P30273; P15941; P18825; P41240; Q9NS75; Q9H4M7; P01034; Q14315; O00592; Q08ET2; O95183; P98198; Q14184; P01889; P10321; P01619; P01834; O60503; O43294; P49771; Q13308; P07204; Q01629; P11215; Q9BSW7; A0A0C4DH55; P41221; P10747; P02751; O60437; Q9H2A7; O94907; Q8IUC4; Q6PJF5; P01705; P21980; P55287; Q8IWU5; Q5GJ75; Q6NZY7; P08571; P01871; P01763; O75084; P56746; Q5KU26; O15197; Q86XX4; P14174; Q99959; P0DMV9; P0DMV8; Q8TBX8; P21730; P01601; Q08116; P01137; Q9Y624; Q86VI4; P54852; P05412; P80723; P17927; P01602; Q9UGI6; P22223; P16144; A0A075B6N2; P41220; A0A087WSY6; P78509; Q13421; Q6UXD5; Q8WVN6; P14415; P01780; P31431; P22735; P05109; P01764; P01614; A0A087WW87; P04433; P02787; P49703; A0A0C4DH25; P0C0L4; P0C0L5; Q8N126; P23083; A0A075B6J9; P01624; P04430; A0A0C4DH72; P06312; P01703; A0A075B6R9; P0CG04; A0A075B6K6; P01717; P0DOY3; P01772; A0A0C4DH68; P0DOY2; A2NJV5 |
| Uniprot | Extracellular exosome | 146 | 1.828579 | 1.66E-13 | P30154; P00450; P80108; P22352; P0CG39; P0DTE7; P0DTE8; P0DUB6; O00469; Q04446; P48960; P62249; Q9NR34; Q06323; P29350; P62888; P51571; P11217; P62316; O95716; P62277; P04216; Q5XXA6; P13798; O60262; Q9NZH0; P00492; Q9UKJ1; Q24JP5; Q6PL18; P01024; P00750; P28062; P15291; Q10588; O43598; Q4ZG55; P62195; Q14393; Q9H3S7; P61353; P39019; P15880; P05155; P09211; P14923; P08123; P12268; Q8TCD5; Q10589; Q9BV40; Q05193; O95833; P17252; P04156; Q99835; Q9UNN8; P13639; P30530; Q9Y230; Q5T9L3; Q96FZ7; P46782; P25685; Q8IV36; P60842; Q96H40; Q9NQX7; Q9Y5Y7; P15941; P41240; P18065; Q9NR99; O75901; P01034; Q16658; P01859; O00592; Q96F10; O95183; P01889; P10321; P01619; P01834; P17050; Q13113; Q14314; Q15113; O95336; P10643; O00391; P03973; P11215; Q08380; P04004 Q641Q3; P01033; P41221; P02751; O60437; Q8WUT4; Q8NCH0; P21980; Q15274; P55287; P22087; P08571; P01871; P01876; Q14117; P62244; Q9P227; P14174; P21810; P0DMV9; P0DMV8; Q8TBX8; P23219; Q9Y624; P80723; P62280; P17927; P01602; P29373; P16144; Q8WVN6; P01780; P31431; P22735; P05109; P01764; P01614; P01861; P04433; P02787; O14745; P0C0L4; P0C0L5; Q9Y5R2; P01857; P01877; P01860; P01591; P0CG04; P0DOY3; P0DOY2 |
| GO | Extracellular vesicular exosome | 146 | 1.863001 | 3.75E-14 | PPP2R1B; CP; GPLD1; GPX3; POTEJ; AMY1B; AMY1C; AMY1A; PLOD2; GBE1; ADGRE5; RPS16; MAN1C1; PSME1; PTPN6; RPL30; SSR4; PYGM; SNRPD2; RAB3D; RPS13; THY1; ANO1; APEH; GNG7; GPRC5B; HPRT1; PILRA; TMEM132A; ATAD2; C3; PLAT; PSMB8; B4GALT1; BST1; DNPH1; GREB1; PSMC5; GAS6; PTPN23; RPL27; RPS19; RPS2; SERPING1; GSTP1; JUP; COL1A2; IMPDH2; NT5C; BST2; VAMP8; DNM1; CLIC3; PRKCA; PRNP; SMO; PROCR; EEF2; AXL; RUVBL2; WLS; CHMP6; RPS5; DNAJB1; HID1; EIF4A1; ZNF486; ITM2C; LYVE1; MUC1; CSK; IGFBP2; MXRA5; RASSF9; CST3; FSCN1; IGHG2; PODXL; SAT2; VAMP5; HLA-B; HLA-C; IGKV3-20; IGKC; NAGA; PDZK1IP1; FGL2; PCOLCE; PGLS; C7; QSOX1; SLPI; ITGAM; LGALS3BP; VTN; METRNL; TIMP1; WNT5A; FN1; PPL; LRRN4; CHST14; TGM2; QPRT; CDH11; FBL; CD14; IGHM; IGHA1; DPYS; RPS15A; ARHGAP23; MIF; BGN; HSPA1B; HSPA1A; PIP4K2C; PTGS1; F11R; BASP1; RPS11; CR1; IGKV1-5; CRABP2; ITGB4; SECTM1; IGHV3-7; SDC4; TGM1; S100A8; IGHV3-23; IGKV2D-40; IGHG4; IGKV3-11; TF; SLC9A3R1; C4A; C4B; MMP24; IGHG1; IGHA2; IGHG3; JCHAIN; IGLC1; IGLC3; IGLC2 |
| GO | Extracellular space | 129 | 2.08141 | 4.27E-16 | LHB; CP; GPLD1; GPX3; SCUBE2; ADAMTS5; POTEJ; GHR; AMY1B; AMY1C; AMY1A; FGF2; CSH1; CLEC11A; IGFBP6; IGLV5-45; COL5A1; CCL14; CCL15; LY86; GPRC5B; C3; PLAT; B4GALT1; CPZ; FJX1; GAS6; SERPING1; GSTP1; ALOX5; COL1A2; SSC5D; PROCR; AXL; NENF; ABI3BP; LXN; SEMA3B; TGFBR3L; MUC1; IGFBP2; CST3; IGHG2; IL34; PODXL; IGKV3-20; IGKC; C1QL1; FGL2; PCOLCE; FLT3LG; QSOX1; THBD; CBLN4; PDGFB; SLPI; COL14A1; ITGAM; LGALS3BP; IGKV3D-7; OLFML3; VTN; METRNL; TIMP1; WNT5A; FN1; CXCL16; DKK1; IGLV2-23; COL1A1; CTSK; SULF2; CD14; IGHM; IGHA1; PTGIS; COLEC12; MIF; BGN; ANGPTL7; IGFBP4; IGKV1D-16; TGFB1; IGKV3OR2-268; LRRTM1; CPXM1; CR1; IGKV1-5; PAPPA; OLFML1; CXCL10; IGKV3D-15; RELN; MSLN; SECTM1; S100A8; IGHV3-23; IGKV2D-40; IGKV2-40; IGHG4; IGKV3-11; CCBE1; TF; CCL2; IGKV3D-20; TPSB2; TPSAB1; C4A; C4B; IGHG1; IGHA2; IGLV2-18; IGHG3; IGKV3-15; CCL21; IGKV1-16; IGKV1-6; EGFL6; IGKV4-1; JCHAIN; IGLV1-40; IGKV2D-24; IGLC1; IGLV4-3; IGLV3-25; IGLC3; IGKV2-24; IGLC2; IGKV2-29 |
| GO | Extracellular region | 126 | 1.760458 | 1.45E-10 | LHB; CP; GPLD1; GPX3; SCUBE2; ADAMTS5; PLGLB1; BAGE4; BAGE3; BAGE2; BAGE5; BAGE; GHR; DEFB128; FGF2; CSH1; ADAMTS18; LNPEP; NHLRC3; CLEC11A; IGFBP6; PTPN6; COL5A1; EPHB2; NTN3; ST3GAL2; THY1; APEH; GMFG; ADAMTS7; C3; PLAT; TRAPPC1; BST1; GAS6; MZB1; SERPING1; CNN2; GSTP1; JUP; ALOX5; COL1A2; IMPDH2; CLSTN1; METTL24; PROCR; EEF2; SMPD3; COL6A6; ABI3BP; IGFBP2; MXRA5; CST3; IGHG2; IL34; IGKV3-20; IGKC; FGL2; PCOLCE; C7; FLT3LG; QSOX1; CBLN4; PDGFB; SLPI; COL14A1; LGALS3BP; SVBP; VTN; TIMP1; WNT5A; FN1; CXCL16; DKK1; IGLV2-23; EMILIN2; COL1A1; CTSK; CD14; IGHV3-48; IGHA1; CA11; MDK; EPHB6; MIF; BGN; ANGPTL7; IGFBP4; PYCARD; HSPA1B; HSPA1A; IGKV1D-16; TGFB1; IGKV1-5; PAPPA; CXCL10; RELN; MSLN; IGHV3-7; S100A8; IGHV3-23; IGKV2D-40; IGHG4; IGKV3-11; TF; CCL2; IGKV3D-20; TPSAB1; C4A; C4B; IGHV1-2; IGHG1; IGHA2; IGHG3; IGKV3-15; CCL21; IGKV1-16; IGKV4-1; JCHAIN; IGLV1-40; IGLC1; IGLV3-25; IGLC3; IGHV3-33; IGLC2; IGKV2-29 |
| FR | Exosomes | 125 | 1.559493 | 1.4E-07 | PPP2R1B; POTEM; CP; NQO1; GPX3; AMY1B; AMY1C; AMY1A; PLOD2; GBE1; ADGRE5; RPS16; PSME1; PTPN6; RPL30; SSR4; EPHB2; FAM65A; SNRPD2; RAB3D; HIST4H4; RPS13; THY1; ANO1; APEH; GNG7; GPRC5B; HPRT1; PILRA; ATAD2; C3; HIST2H4B; HIST2H4A; PLAT; PSMB8; B4GALT1; DNPH1; GREB1; PSMC5; PTPN23; RPL27; RPS19; RPS2; ATP5I; SERPING1; GSTP1; JUP; IMPDH2; NT5C; BST2; VAMP8; CLSTN1; CLIC3; PRKCA; ASB6; PRNP; SMO; EEF2; RUVBL2; CHMP6; RPS5; DNAJB1; EIF4A1; ZNF486; ITM2C; ASNA1; MUC1; CSK; MXRA5; RASSF9; CST3; FLNC; FSCN1; PODXL; SAT2; SNRPD1; VAMP5; HLA-B; HLA-C; PDZK1IP1; FGL2; PGLS; SLC25A6; QSOX1; IFITM2; SLPI; LGALS3BP; VTN; METRNL; FN1; PPL; PTGFRN; CHST14; HIST1H4L; TGM2; QPRT; FBL; CD14; DPYS; RPS15A; ARHGAP23; MIF; BGN; HSPA1B; HSPA1A; PIP4K2C; PTGS1; TGFB1; F11R; BASP1; RPS11; CR1; CRABP2; ITGB4; RELN; SECTM1; SDC4; TGM1; S100A8; TF; SLC9A3R1; C4A; C4B; MMP24; JCHAIN |
| FR | Extracellular | 108 | 1.50837 | 5.96E-06 | LHB; CP; GPLD1; GPX3; SCUBE2; ADAMTS5; NOV; GHR; NOTCH2NL; AMY1B; AMY1C; AMY1A; FGF2; ZBTB16; CSH1; ADAMTS18; LNPEP; PLOD2; CLEC11A; TRADD; IGFBP6; SQLE; COL5A1; NTN3; PYGM; CCL14; CCL15; LY86; STX10; GNG7; ADAMTS7; C3; CYC1; PLAT; B4GALT1; BST1; CPZ; GAS6; ATP5I; BCAT2; SERPING1; APLP1; JUP; COL1A2; PRNP; CKAP2; AXL; NENF; SEMA3B; MUC1; IGFBP2; CST3; HLA-B; ZMYM3; NAGA; FGL2; PCOLCE; C7; FLT3LG; QSOX1; THBD; PDGFB; SLPI; COL14A1; LGALS3BP; OLFML3; VTN; RPS27; JUND; TIMP1; WNT5A; FN1; CXCL16; DKK1; EMILIN2; TGM2; COL1A1; CTSK; CDH11; SULF2; CD14; CA11; MDK; FRAS1; MIF; BGN; ANGPTL7; IGFBP4; TGFB1; BASP1; CDH3; PAPPA; RGS2; CXCL10; RELN; SECTM1; TGM1; S100A8; CCBE1; TF; CCL2; TPSB2; TPSAB1; C4A; EGR1; CCL21; EGFL6; JCHAIN |
| GO | Cell surface | 41 | 1.809418 | 0.000195 | SCUBE2; GHR; DMD; EPHB2; NLGN2; THY1; GPRC5B; ADAMTS7; C3; PLAT; LAYN; BST2; CLSTN1; PRNP; PROCR; AXL; ROR1; SLC46A1; TGFBR3L; FCER1G; VAMP5; HLA-B; HLA-C; FLT3LG; THBD; PDGFB; ITGAM; WNT5A; CD28; PTGFRN; SULF2; IGHM; MIF; BGN; TGFB1; LRRTM1; CR1; ITGB4; MSLN; SDC4; TF |
| GO | External side of plasma membrane | 37 | 2.257822 | 3.69E-06 | BTNL9; IGHV3-38; GHR; IGHV1OR21-1; THY1; STAB2; B4GALT1; CLEC2B; ASTN1; FCER1G; IGHG2; IGKC; THBD; ITGAM; CD28; CTSK; CD14; IGHM; IGHV3-48; IGHA1; CXCL10; ATP1B2; IGHV3-7; IGHV3-23; IGHG4; TRDC; IGHV1-18; IGHV4-31; IGHV1-2; IGHG1; IGHA2; IGHG3; IGHV3-15; IGLC1; IGLC3; IGHV3-33; IGLC2 |
| GO | Focal adhesion | 35 | 2.211368 | 1.06E-05 | CD99; FHL1; NEXN; ARHGAP24; ADGRE5; RPS16; RPL30; GRB7; RPS13; THY1; RPL27; RPS19; RPS2; CNN2; RPL19; JUP; LAYN; MRC2; SCARF2; HCK; PROCR; SORBS3; RPS5; ZNF185; ZYX; FLNC; TGFB1I1; PTK7; FHL3; TGM2; HSPA1B; HSPA1A; RPS11; ITGB4; SDC4 |
| GO | Blood microparticle | 34 | 6.414055 | 2E-18 | CP; C3; PSMC5; SERPING1; IGHG2; IGKV3-20; IGKC; LGALS3BP; VTN; FN1; IGHM; IGHA1; HSPA1B; HSPA1A; TGFB1; IGKV1-5; IGHV3-7; IGHV3-23; IGKV2D-40; IGHG4; IGKV3-11; TF; C4A; C4B; IGHG1; IGHA2; IGHG3; IGKV3-15; IGKV4-1; JCHAIN; IGLC1; IGLV3-25; IGLC3; IGLC2 |
| FR | Extracellular space | 28 | 1.766974 | 0.002611 | CP; GPX3; GHR; FGF2; CCL14; CCL15; FJX1; GAS6; COL1A2; EFNA5; ABI3BP; CST3; IL34; PCOLCE; QSOX1; COL14A1; LGALS3BP; VTN; WNT5A; FN1; CXCL16; COL1A1; SULF2; TGFB1; SECTM1; CCL2; TPSAB1; EGFL6 |
| Uniprot | Collagen-containing extracellular matrix | 25 | 1.907397 | 0.001622 | Q9UNA0; P20908; P05155; P08123; A1L4H1; A6NMZ7; Q7Z7G0; Q13214; Q9NR99; Q14314; Q9NTU7; P01127; P03973; Q05707; Q08380; P04004; P02751; Q9BXX0; P21980; P02452; P21810; O43827; P01137; P05109; P20231 |
| GO | Extracellular matrix | 20 | 2.178408 | 0.000965 | GPLD1; ADAMTS5; ADAMTS18; COL5A1; ADAMTS7; COL1A2; SSC5D; COL6A6; COL14A1; TIMP1; FN1; TGM2; COL1A1; COLEC12; BGN; TGFB1; LRRTM1; RELN; CCBE1; MMP24 |
| GO | Membrane raft | 16 | 1.816795 | 0.016038 | PPP2R1B; DMD; TRADD; THY1; ANO1; BIRC3; CLN6; BST2; CLIP3; PRNP; CSK; PODXL; CD14; PLLP; SDC4; SLC9A3R1 |
| GO | Cell-cell junction | 12 | 1.738572 | 0.04515 | USP53; LCP2; PTPN6; CNN2; JUP; VAV1; CSK; FSCN1; PTK7; PKP2; F11R; CADM3 |
| GO | Collagen | 6 | 2.682229 | 0.024079 | COL6A6; C1QL1; COL14A1; EMILIN2; COLEC12; CCBE1 |
| Uniprot | Collagen trimer | 6 | 2.635178 | 0.025979 | A6NMZ7; O75973; Q05707; Q9BXX0; Q5KU26; Q6UXH8 |
| Uniprot | Anchored component of external side of plasma membrane | 4 | 5.272984 | 0.006164 | P04216; P04156; P52803; P08571 |
| GO | Anchored to external side of plasma membrane | 4 | 5.367132 | 0.005792 | THY1; PRNP; EFNA5; CD14 |
| GO | Desmosome | 4 | 4.294135 | 0.013052 | B4GALT1; JUP; PPL; PKP2 |
| GO | Extracellular matrix structural constituent | 12 | 1.801802 | 0.004447716 | COL5A1; COL1A2; ABI3BP; MXRA5; FGL2; PCOLCE; COL14A1; VTN; FN1; COL1A1; FRAS1; BGN |
| GO | Heparin binding | 11 | 1.651652 | 1.783513 | ADAMTS5; FGF2; COL5A1; CCL15; APLP1; LXN; PCOLCE; VTN; FN1; MDK; CXCL10 |
| GO | Collagen binding | 7 | 1.051051 | 0.009339819 | MRC2; PCOLCE; PDGFB; COL14A1; FN1; CTSK; CCBE1 |
| GO | Extracellular matrix structural constituent conferring tensile strength | 5 | 0.750751 | 0.016196417 | COL5A1; COL1A2; COL6A6; COL14A1; COL1A1 |
| GO | Extracellular matrix binding | 4 | 0.600601 | 0.022744862 | ADAMTS5; SSC5D; VTN; BGN |
| GO | Fibronectin binding | 4 | 0.600601 | 0.025381594 | IGFBP6; SSC5D; CTSK; SDC4 |
| GO | Cell adhesion | 29 | 1.618089 | 0.00776 | PCDH9; FER; ADGRE5; COL5A1; STAB2; B4GALT1; APLP1; CLSTN1; PRKCA; HCK; PRKD2; SORBS3; COL6A6; PODXL; SIGLEC14; TGFB1I1; PTK7; ITGAM; LGALS3BP; VTN; FN1; CDH11; CLDN15; CDH3; ITGB4; RELN; MSLN; CCL2; EGFL6 |
| GO | Extracellular matrix assembly | 6 | 14.73656 | 9.95E-07 | GAS6; FKBP10; COL1A2; SMPD3; QSOX1; TGFB1 |
| GO | Cellular response to extracellular stimulus | 3 | 4.274579 | 0.031507 | CDKN2B; AXL; FOS |
| GO | Positive regulation of cartilage development | 3 | 4.777176 | 0.023349 | ZBTB16; WNT5A; MDK |
| FR | Cell junction organization | 10 | 3.061558 | 0.001385 | ARHGEF6; JUP; FLNC; PVRL2; CDH11; CLDN15; F11R; CDH3; ITGB4; CADM3 |
| FR | Cell surface interactions at the vascular wall | 9 | 2.640903 | 0.006543 | PIK3R2; PTPN6; GRB7; GAS6; FCER1G; THBD; ITGAM; FN1; F11R |
| FR | Beta3 integrin cell surface interactions | 8 | 3.930824 | 0.000785 | THY1; COL1A2; PDGFB; VTN; FN1; COL1A1; F11R; SDC4 |
| Reactome | ECM proteoglycans | 8 | 2.538666 | 0.013199 | P20908; P08123; A6NMZ7; P04004; P02751; P02452; P21810; P01137 |
| FR | Integrin cell surface interactions | 8 | 2.347795 | 0.019814 | COL1A2; CSK; ITGAM; VTN; FN1; COL1A1; F11R; ITGB4 |
| FR | Cell-cell junction organization | 7 | 3.287226 | 0.004802 | JUP; PVRL2; CDH11; CLDN15; F11R; CDH3; CADM3 |
| Reactome | Collagen biosynthesis and modifying enzymes | 7 | 2.520125 | 0.020614 | O00469; P20908; P08123; A6NMZ7; Q15113; Q05707; P02452 |
| Reactome | Non-integrin membrane-ECM interactions | 7 | 4.220785 | 0.001149 | P11532; P09038; P20908; P08123; P01127; P02751; P02452 |
| Reactome | Assembly of collagen fibrils and other multimeric structures | 6 | 2.895085 | 0.01675 | P20908; P08123; A6NMZ7; Q05707; P02452; P16144 |
| Reactome | Collagen degradation | 6 | 2.261884 | 0.048941 | P20908; P08123; A6NMZ7; Q05707; P02452; P43235 |
| FR | Adherens junctions interactions | 5 | 3.915017 | 0.007884 | JUP; PVRL2; CDH11; CDH3; CADM3 |
| FR | Beta2 integrin cell surface interactions | 5 | 3.775245 | 0.009234 | THY1; C3; PLAT; ITGAM; F11R |
| Reactome | Collagen chain trimerization | 5 | 2.742395 | 0.034606 | P20908; P08123; A6NMZ7; Q05707; P02452 |
| Reactome | Activation of Matrix Metalloproteinases | 4 | 2.92646 | 0.046334 | P01033; P43235; Q15661; Q9Y5R2 |
| Reactome | Extracellular matrix organization | 4 | 6.435872 | 0.002775 | P20908; P08123; P02751; P02452 |
