# Supplementary material for: Understanding the role of visceral fat in metabolically healthy versus unhealthy obesity: a sex-based analysis of the transcriptome
Source: Biol Sex Differ. 2025 Nov 6;16:92. doi: 10.1186/s13293-025-00777-6 (PMC12593901; doi:10.1186/s13293-025-00777-6)
Supplement: Supplementary file 7 — Additional file 7. [file 13293_2025_777_MOESM7_ESM.docx]

| **Supplementary table S7. Functional enrichment analysis based on the related extracellular component of the differential transcript expression of the MU female vs. MU male** | | | | | |
| --- | --- | --- | --- | --- | --- |
| **Database** | **Related extracellular component** | **No. of genes** | **Fold enrichment** | ***p*-value** | **Genes** |
| GO | Heparin binding | 6 | 2.947147 | 0.016821 | PRELP; VEGFB; CLEC3B; THBS4; SFRP1; FURIN |
| GO | Integrin binding | 5 | 2.696054 | 0.039053 | THBS4; ITGA11; FERMT3; DST; CD9 |
| GO | Hyaluronic acid binding | 2 | 6.404651 | 0.038952 | USP17L6P; USP17L24 |
