# Supplementary material for: Understanding the role of visceral fat in metabolically healthy versus unhealthy obesity: a sex-based analysis of the transcriptome
Source: Biol Sex Differ. 2025 Nov 6;16:92. doi: 10.1186/s13293-025-00777-6 (PMC12593901; doi:10.1186/s13293-025-00777-6)
Supplement: Supplementary file 8 — Additional file 8. [file 13293_2025_777_MOESM8_ESM.docx]

| **Supplementary table S8. Functional enrichment analysis based on the related inflammatory component of the differential transcript expression of the MH female vs. MH male** | | | | | | |
| --- | --- | --- | --- | --- | --- | --- |
| **Database** | **Related inflammatory**  **Component** | **No. of genes** | **Fold enrichment** | ***p*-value** | **Genes** |  |
| GO | Innate immune response | 53 | 2.537348 | 4.86E-10 | LGR4; TRIM51; MORC3; IGHV3-38; DEFB128; IGHV1OR21-1; FER; LY86; SLC15A3; ELF4; SERPING1; TNFAIP8L2; BST2; SSC5D; TRIM21; HCK; AXL; ECSIT; FCER1G; IGHG2; IL34; HLA-B; HLA-C; IGKC; SLPI; ITGAM; TRIM28; CD14; IGHM; IGHV3-48; IGHA1; COLEC12; MIF; PYCARD; IGHV3-7; S100A8; IGHV3-23; IGHG4; TRDC; C4A; C4B; IGHV1-18; IGHV4-31; IGHV1-2; IGHG1; IGHA2; IGHG3; IGHV3-15; JCHAIN; IGLC1; IGLC3; IGHV3-33; IGLC2 |  |
| GO | Immune response | 50 | 3.082349 | 1.42E-12 | ADGRE5; LCP2; PIK3R2; IGLV5-45; C3; VAV1; CRIP1; CYSLTR2; HLA-B; HLA-C; IGKV3-20; IGKC; IFITM2; SLPI; IGKV3D-7; VTN; IGLV2-23; CTSK; IGHV3-48; IGHA1; C5AR1; IGKV1D-16; RGS1; IGKV3OR2-268; IGKV1-5; IGKV3D-15; SECTM1; IGHV3-7; IGHV3-23; IGKV2D-40; IGKV2-40; IGKV3-11; IGKV3D-20; IGHV1-2; IGHA2; IGLV2-18; IGKV3-15; CCL21; IGKV1-16; IGKV1-6; IGKV4-1; JCHAIN; IGLV1-40; IGKV2D-24; IGLC1; IGLV4-3; IGLV3-25; IGHV3-33; IGKV2-24; IGKV2-29 |  |
| GO | Adaptive immune response | 41 | 2.580663 | 2.98E-08 | IGLV5-45; TRBJ2-4; PRKD2; CSK; IGHG2; HLA-B; HLA-C; IGKV3-20; IGKC; IGKV3D-7; IGLV2-23; IGHM; IGHA1; IGKV1D-16; IGKV1-5; TRBV20-1; IGKV3D-15; IGKV2D-40; IGKV2-40; IGHG4; IGKV3-11; IGKV3D-20; TRDC; IGHG1; IGHA2; IGLV2-18; IGHG3; IGKV3-15; IGKV1-16; IGKV1-6; IGKV4-1; JCHAIN; IGLV1-40; IGKV2D-24; IGLC1; IGLV4-3; IGLV3-25; IGLC3; IGKV2-24; IGLC2; IGKV2-29 |  |
| GO | Inflammatory response | 27 | 1.864858 | 0.001493 | ADGRE5; PTGER2; GGT5; CCL14; CCL15; LY86; C3; HCK; AXL; BLNK; LXN; IL34; CD14; MIF; IGFBP4; PYCARD; PTGS1; C5AR1; TGFB1; F11R; CXCL10; S100A8; FOS; CCL2; C4A; C4B; CCL21 |  |
| GO | Immunoglobulin receptor binding | 23 | 3.453453 | 6.846727 | IGHV3-38; IGHV1OR21-1; IGHG2; IGKC; IGHM; IGHV3-48; IGHA1; IGHV3-7; IGHV3-23; IGHG4; TRDC; IGHV1-18; IGHV4-31; IGHV1-2; IGHG1; IGHA2; IGHG3; IGHV3-15; JCHAIN; IGLC1; IGLC3; IGHV3-33; IGLC2 |  |
| GO | Immunoglobulin production | 23 | 6.211296 | 1.4E-12 | IGLV5-45; IGKV3-20; IGKV3D-7; IGLV2-23; IGKV1D-16; IGKV3OR2-268; IGKV1-5; IGKV3D-15; IGKV2D-40; IGKV2-40; IGKV3-11; IGKV3D-20; IGLV2-18; IGKV3-15; IGKV1-16; IGKV1-6; IGKV4-1; IGLV1-40; IGKV2D-24; IGLV4-3; IGLV3-25; IGKV2-24; IGKV2-29 |  |
| GO | Immunoglobulin complex | 21 | 6.251443 | 1.16E-11 | IGLV5-45; IGKV3D-7; IGLV2-23; IGKV1D-16; IGKV1-5; IGKV3D-15; IGKV2D-40; IGKV2-40; IGKV3-11; IGKV3D-20; IGLV2-18; IGKV3-15; IGKV1-16; IGKV1-6; IGKV4-1; IGLV1-40; IGKV2D-24; IGLV4-3; IGLV3-25; IGKV2-24; IGKV2-29 |  |
| Uniprot | Immunoglobulin complex. circulating | 21 | 7.273015 | 4.56E-13 | A0A0C4DH36; P01859; P01834; P01871; P01763; P01876; P01780; P01764; P01861; B7Z8K6; A0A0C4DH31; P0DP07; P23083; P01857; P01877; P01860; A0A0B4J1V0; P0CG04; P0DOY3; P01772; P0DOY2 |  |
| GO | Extracellular matrix organization | 13 | 2.181391 | 0.00698 | ADAMTS5; ADAMTS18; COL5A1; ADAMTS7; B4GALT1; APLP1; COL1A2; COL6A6; ABI3BP; COL14A1; COL1A1; MMP24; EGFL6 |  |
| GO | Positive regulation of angiogenesis | 10 | 1.972381 | 0.031257 | FGF2; C3; JUP; PRKCA; PRKD2; WNT5A; EMILIN2; PTGIS; C5AR1; CCBE1 |  |
| GO | Igg immunoglobulin complex | 9 | 21.91707 | 6.9E-12 | IGHG2; IGKC; IGHA1; IGHG4; IGHG1; IGHG3; IGLC1; IGLC3; IGLC2 |  |
| GO | Cellular response to interleukin-1 | 9 | 3.040112 | 0.002764 | CCL14; CCL15; ADAMTS7; PTGIS; PYCARD; NR1D1; KLF2; CCL2; CCL21 |  |
| GO | Response to lipopolysaccharide | 9 | 1.91512 | 0.046544 | FER; PTGER2; IL10RA; THBD; SLPI; JUND; NR4A1; S100A8; FOS |  |
| GO | Negative regulation of inflammatory response | 8 | 2.184049 | 0.030526 | TNFAIP8L2; ALOX5; FEM1A; METRNL; PTGIS; KLF4; NR1D1; ZFP36 |  |
| GO | Iga immunoglobulin complex | 6 | 20.09495 | 6.97E-08 | IGKC; IGHA1; IGHA2; IGLC1; IGLC3; IGLC2 |  |
| GO | Positive regulation of interleukin-8 production | 6 | 2.616506 | 0.026854 | PRKD2; WNT5A; CD14; PYCARD; HSPA1B; HSPA1A |  |
| FR | IL6-mediated signaling events | 6 | 2.69839 | 0.022482 | VAV1; HCK; TIMP1; JUN; FOS; JUNB |  |
| GO | Igm immunoglobulin complex | 5 | 19.141 | 1.41E-06 | IGKC; IGHM; IGLC1; IGLC3; IGLC2; |  |
| GO | Igd immunoglobulin complex | 4 | 15.32044 | 6.16E-05 | IGKC; IGLC1; IGLC3; IGLC2 |  |
| GO | Ige immunoglobulin complex | 4 | 15.32044 | 6.16E-05 | IGKC; IGLC1; IGLC3; IGLC2 |  |
| GO | Monomeric iga immunoglobulin complex | 4 | 26.78212 | 1.93E-06 | IGKV3-20; IGHA1; IGHA2; JCHAIN |  |
| GO | Secretory iga immunoglobulin complex | 4 | 21.43639 | 9.35E-06 | IGKV3-20; IGHA1; IGHA2; JCHAIN |  |
| GO | Peptide antigen binding | 4 | 0.600601 | 2.888338 | DHCR24; MAML1; HLA-B; HLA-C |  |
| GO | Response to interferon-gamma | 4 | 4.704752 | 0.009424 | BST2; TRIM21; IFITM2; CXCL16 |  |
| FR | IL12 signaling mediated by STAT4 | 4 | 2.917542 | 0.046094 | CD28; TGFB1; JUN; FOS |  |
| GO | Pentameric igm immunoglobulin complex | 3 | 26.78212 | 5.18E-05 | IGKV3-20; IGHM; JCHAIN |  |
| GO | Secretory dimeric iga immunoglobulin complex | 3 | 26.78212 | 5.18E-05 | IGHA1; IGHA2; JCHAIN |  |
| GO | Cell migration involved in sprouting angiogenesis | 3 | 3.86767 | 0.04096 | GPLD1; FGF2; NR4A1 |  |
| GO | Desmosome assembly | 3 | 20.26428 | 0.000197 | JUP; PRKCA; PKP2 |  |
| Reactome | Crosslinking of collagen fibrils | 3 | 4.026211 | 0.036451 | P08123; Q15113; P02452 |  |
