# Supplementary material for: Understanding the role of visceral fat in metabolically healthy versus unhealthy obesity: a sex-based analysis of the transcriptome
Source: Biol Sex Differ. 2025 Nov 6;16:92. doi: 10.1186/s13293-025-00777-6 (PMC12593901; doi:10.1186/s13293-025-00777-6)
Supplement: Supplementary file 9 — Additional file 9. [file 13293_2025_777_MOESM9_ESM.docx]

| **Supplementary table S9. Functional enrichment analysis based on the related inflammatory component of the differential transcript expression of the MU female vs. MU male** | | | | | |
| --- | --- | --- | --- | --- | --- |
| **Database** | **Related inflammatory**  **Component** | **No. of genes** | **Fold enrichment** | ***p*-value** | **Genes** |
| GO | Antigen binding | 7 | 4.120101 | 0.001633 | IGHM; IGHV4-31; IGHA1; IGHV3-66; IGLV6-57; IGLL5; MFAP4 |
| GO | Immunoglobulin receptor binding | 5 | 4.512762 | 0.005137 | IGHM; IGHV4-31; IGHA1; IGHV3-66; IGLL5 |
| GO | B-cell receptor signaling pathway | 5 | 3.321913 | 0.01783 | IGHM; IGHV4-31; IGHA1; IGHV3-66; IGLL5 |
| GO | Cellular response to tumor necrosis factor | 5 | 3.376366 | 0.016735 | CCL4L1; SFRP1; CIB1; BRCA1; YBX3 |
| GO | Phagocytosis. Engulfment | 5 | 3.710931 | 0.011505 | IGHM; IGHV4-31; IGHA1; IGHV3-66; IGLL5 |
| GO | Phagocytosis. Recognition | 5 | 4.335864 | 0.006074 | IGHM; IGHV4-31; IGHA1; IGHV3-66; IGLL5 |
| GO | Positive regulation of angiogenesis | 5 | 3.006718 | 0.026111 | VEGFB; RRAS; HSPB6; ACVRL1; BRCA1 |
| GO | Positive regulation of B-cell activation | 5 | 4.429098 | 0.005555 | IGHM; IGHV4-31; IGHA1; IGHV3-66; IGLL5 |
