# Supplementary material for: Understanding the role of visceral fat in metabolically healthy versus unhealthy obesity: a sex-based analysis of the transcriptome
Source: Biol Sex Differ. 2025 Nov 6;16:92. doi: 10.1186/s13293-025-00777-6 (PMC12593901; doi:10.1186/s13293-025-00777-6)
Supplement: Supplementary file 10 — Additional file 10. [file 13293_2025_777_MOESM10_ESM.docx]

| **Supplementary Table S10. Common related extracellular components and their respective transcripts of the MH female vs. MU female and MH male vs. MU male** | | | |
| --- | --- | --- | --- |
| **Database** | **Related extracellular component** | **MH female vs. MU female** | **MH male vs. MU male** |
| GO | Plasma membrane | IGKV4-1; IGHV1-2; IGHG1; IGLV2-18; IGKV3D-15; IGKV2-40; IGKV3D-20; IGHM; IGHV3-7; IGKV2D-40; RGS2; IGKV3D-7; IGKC; IGKV1-5; HLA-B; HLA-C; FRMD8P1; RELN; FN1; **JUN**; STAB2; SRR; PALM2; ARL17A; OR7D2; ANGPT1; PTGER3; STS; RNF157; SLC19A3; LHCGR; ADAM12; TMEM170B | ZBTB16; MALL; CD99; ADGRF5; BST1; ANXA3; TNFAIP8L3; ATP2B1; DPP4; KRT19; EMP3; RHPN2; PROCR; GAS1; SGMS2; AHNAK2; PKP2; PTPN3; CDON; PERP; **JUN**; HSPA2; GJA1; PRKCI; AQP9; CD9; SYT17; CD200; SGK1; BCHE; DSP; DSC3; SDC4; RGS1; PPL; CLDN1 |
| GO | Extracellular space | IGKV4-1; IGHG1; IGLV2-18; IGKV3D-15; IGKV2-40; IGKV3D-20; IGHM; IGKV2D-40; OLFM2; IGKV3OR2-268; IGKV3D-7; IGKC; IGKV1-5; RELN; FN1; SERPINI1; COL1A1; ANGPT1; PRELP; SCUBE2; GPLD1 | EDN1; FBN1; GUSBP1; THBS1; BMP4; TIMP1; FGL2; PDGFD; PROCR; ITLN1; CD9; OGN; BCHE; FGF9; CSN1S1; PAPPA; CCBE1; PRG4; CCL2; EGFL6 |
| GO | Extracellular region | IGKV4-1; IGHV1-2; IGHG1; IGKV3D-20; IGHV3-7; IGKV2D-40; OLFM2; MDK; IGKC; IGKV1-5; RELN; C1orf54; FN1; COL1A1; ANGPT1; PRELP; SCUBE2; ADAMTS12; ADAM12; GPLD1 | EDN1; FBN1; THBS1; BST1; DPP4; BMP4; TIMP1; FGL2; PDGFD; PROCR; ITLN1; WFDC8; RSPO1; OGN; BCHE; FGF9; CSN1S1; PAPPA; DSC3; CCL2 |
| Uniprot | Extracellular exosome | P01857; P01871; P01780; P01614; P01834; P01602; P01889; P10321; P02751; Q99574; Q969L2; Q15389; P51888; P30711; P80108 | Q96RW7; P07996; Q9P0V3; Q10588; P05783; P12429; P20020; P27487; P01033; P08727; Q14314; Q9UNN8; Q8WWA0; Q9Y5R2; P54652; P41743; P21926; P20774; P15924; P31431; O60437 |
| GO | Extracellular vesicular exosome | IGHG1; IGHM; IGHV3-7; IGKV2D-40; IGKC; IGKV1-5; HLA-B; HLA-C; FN1; SERPINI1; MAL2; ANGPT1; PRELP; GSTT1; GPLD1 | HMCN1; THBS1; SH3BP4; BST1; KRT18; ANXA3; ATP2B1; DPP4; TIMP1; KRT19; FGL2; PROCR; ITLN1; MMP24; HSPA2; PRKCI; CD9; OGN; DSP; SDC4; PPL |
| FR | Extracellular | MDK; RGS2; HLA-B; RELN; FN1; RPS27; RBP1; COL1A1; ANGPT1; SCUBE2; ADAMTS12; ADAM12; GPLD1 | ZBTB16; EDN1; FBN1; HMCN1; THBS1; BST1; KRT18; DPP4; BMP4; TIMP1; FGL2; PDGFD; ITLN1; PRKCI; WFDC8; RSPO1; OGN; BCHE; FGF9; DSP; CSN1S1; PAPPA; DSC3; CCBE1; PRG4; CCL2; EGFL6; EGR1 |
| GO | Extracellular matrix | RELN; FN1; COL1A1; PRELP; ADAMTS12; GPLD1 | FBN1; THBS1; TIMP1; MMP24; CCBE1 |
| Uniprot | Collagen-containing extracellular matrix | P02751; P02452; Q15389; P51888 | P35555; Q96RW7; P07996; Q14314; P20774; Q92954 |
| FR | Beta3 integrin cell surface interactions | FN1; COL1A1 | FBN1; THBS1; SDC4 |
