# Supplementary material for: Understanding the role of visceral fat in metabolically healthy versus unhealthy obesity: a sex-based analysis of the transcriptome
Source: Biol Sex Differ. 2025 Nov 6;16:92. doi: 10.1186/s13293-025-00777-6 (PMC12593901; doi:10.1186/s13293-025-00777-6)
Supplement: Supplementary file 11 — Additional file 11. [file 13293_2025_777_MOESM11_ESM.docx]

| **Supplementary Table S11. Common related extracellular components and their respective transcripts of the MH female vs. MU female and MH male vs. MU male** | | |
| --- | --- | --- |
| **Common extracellular component: 9** | | |
|  | **MH female vs MU female** | **MH male vs MU male** |
| Plasma membrane:1 common transcript | ***JUN*** | ***JUN*** |
|  | *IGKV4-1* | *ZBTB16* |
|  | *IGHV1-2* | *MALL* |
|  | *IGHG1* | *CD99* |
|  | *IGLV2-18* | *ADGRF5* |
|  | *IGKV3D-15* | *BST1* |
|  | *IGKV2-40* | *ANXA3* |
|  | *IGKV3D-20* | *TNFAIP8L3* |
|  | *IGHM* | *ATP2B1* |
|  | *IGHV3-7* | *DPP4* |
|  | *IGKV2D-40* | *KRT19* |
|  | *RGS2* | *EMP3* |
|  | *IGKV3D-7* | *RHPN2* |
|  | *IGKC* | *PROCR* |
|  | *IGKV1-5* | *GAS1* |
|  | *HLA-B* | *SGMS2* |
|  | *HLA-C* | *AHNAK2* |
|  | *FRMD8P1* | *PKP2* |
|  | *RELN* | *PTPN3* |
|  | *FN1* | *CDON* |
|  | *STAB2* | *PERP* |
|  | *SRR* | *HSPA2* |
|  | *PALM2* | *GJA1* |
|  | *ARL17A* | *PRKCI* |
|  | *OR7D2* | *AQP9* |
|  | *ANGPT1* | *CD9* |
|  | *PTGER3* | *SYT17* |
|  | *STS* | *CD200* |
|  | *RNF157* | *SGK1* |
|  | *SLC19A3* | *BCHE* |
|  | *LHCGR* | *DSP* |
|  | *ADAM12* | *DSC3* |
|  | *TMEM170B* | *SDC4* |
|  |  | *RGS1* |
|  |  | *PPL* |
|  |  | *CLDN1* |
| Extracellular space:  0 common transcripts | *IGKV4-1* | *EDN1* |
|  | *IGHG1* | *FBN1* |
|  | *IGLV2-18* | *GUSBP1* |
|  | *IGKV3D-15* | *THBS1* |
|  | *IGKV2-40* | *BMP4* |
|  | *IGKV3D-20* | *TIMP1* |
|  | *IGHM* | *FGL2* |
|  | *IGKV2D-40* | *PDGFD* |
|  | *OLFM2* | *PROCR* |
|  | *IGKV3OR2-268* | *ITLN1* |
|  | *IGKV3D-7* | *CD9* |
|  | *IGKC* | *OGN* |
|  | *IGKV1-5* | *BCHE* |
|  | *RELN* | *FGF9* |
|  | *FN1* | *CSN1S1* |
|  | *SERPINI1* | *PAPPA* |
|  | *COL1A1* | *CCBE1* |
|  | *ANGPT1* | *PRG4* |
|  | *PRELP* | *CCL2* |
|  | *SCUBE2* | *EGFL6* |
|  | *GPLD1* |  |
| Extracellular región:  0 common transcripts | *IGKV4-1* | *EDN1* |
|  | *IGHV1-2* | *FBN1* |
|  | *IGHG1* | *THBS1* |
|  | *IGKV3D-20* | *BST1* |
|  | *IGHV3-7* | *DPP4* |
|  | *IGKV2D-40* | *BMP4* |
|  | *OLFM2* | *TIMP1* |
|  | *MDK* | *FGL2* |
|  | *IGKC* | *PDGFD* |
|  | *IGKV1-5* | *PROCR* |
|  | *RELN* | *ITLN1* |
|  | *C1orf54* | *WFDC8* |
|  | *FN1* | *RSPO1* |
|  | *COL1A1* | *OGN* |
|  | *ANGPT1* | *BCHE* |
|  | *PRELP* | *FGF9* |
|  | *SCUBE2* | *CSN1S1* |
|  | *ADAMTS12* | *PAPPA* |
|  | *ADAM12* | *DSC3* |
|  | *GPLD1* | *CCL2* |
| Extracellular exosome:  0 common transcripts | *P01857* | *Q96RW7* |
|  | *P01871* | *P07996* |
|  | *P01780* | *Q9P0V3* |
|  | *P01614* | *Q10588* |
|  | *P01834* | *P05783* |
|  | *P01602* | *P12429* |
|  | *P01889* | *P20020* |
|  | *P10321* | *P27487* |
|  | *P02751* | *P01033* |
|  | *Q99574* | *P08727* |
|  | *Q969L2* | *Q14314* |
|  | *Q15389* | *Q9UNN8* |
|  | *P51888* | *Q8WWA0* |
|  | *P30711* | *Q9Y5R2* |
|  | *P80108* | *P54652* |
|  |  | *P41743* |
|  |  | *P21926* |
|  |  | *P20774* |
|  |  | *P15924* |
|  |  | *P31431* |
|  |  | *O60437* |
| Extracellular vesicular exosome:  0 common transcripts | *IGHG1* | *HMCN1* |
|  | *IGHM* | *THBS1* |
|  | *IGHV3-7* | *SH3BP4* |
|  | *IGKV2D-40* | *BST1* |
|  | *IGKC* | *KRT18* |
|  | *IGKV1-5* | *ANXA3* |
|  | *HLA-B* | *ATP2B1* |
|  | *HLA-C* | *DPP4* |
|  | *FN1* | *TIMP1* |
|  | *SERPINI1* | *KRT19* |
|  | *MAL2* | *FGL2* |
|  | *ANGPT1* | *PROCR* |
|  | *PRELP* | *ITLN1* |
|  | *GSTT1* | *MMP24* |
|  | *GPLD1* | *HSPA2* |
|  |  | *PRKCI* |
|  |  | *CD9* |
|  |  | *OGN* |
|  |  | *DSP* |
|  |  | *SDC4* |
|  |  | *PPL* |
| Extracellular:  0 common transcripts | *MDK* | *ZBTB16* |
|  | *RGS2* | *EDN1* |
|  | *HLA-B* | *FBN1* |
|  | *RELN* | *HMCN1* |
|  | *FN1* | *THBS1* |
|  | *RPS27* | *BST1* |
|  | *RBP1* | *KRT18* |
|  | *COL1A1* | *DPP4* |
|  | *ANGPT1* | *BMP4* |
|  | *SCUBE2* | *TIMP1* |
|  | *ADAMTS12* | *FGL2* |
|  | *ADAM12* | *PDGFD* |
|  | *GPLD1* | *ITLN1* |
|  |  | *PRKCI* |
|  |  | *WFDC8* |
|  |  | *RSPO1* |
|  |  | *OGN* |
|  |  | *BCHE* |
|  |  | *FGF9* |
|  |  | *DSP* |
|  |  | *CSN1S1* |
|  |  | *PAPP* |
|  |  | *DSC3* |
|  |  | *CCBE1* |
|  |  | *PRG4* |
|  |  | *CCL2* |
|  |  | *EGFL6* |
|  |  | *EGR1* |
| Extracellular matrix:  0 common transcripts | *RELN* | *FBN1* |
|  | *FN1* | *THBS1* |
|  | *COL1A1* | *TIMP1* |
|  | *PRELP* | *MMP24* |
|  | *ADAMTS12* | *CCBE1* |
|  | *GPLD1* |  |
| Collagen-containing extracellular matrix:  0 common transcripts | *P02751* | *P35555* |
|  | *P02452* | *Q96RW7* |
|  | *Q15389* | *P07996* |
|  | *P51888* | *Q14314* |
|  |  | *P20774* |
|  |  | *Q92954* |
| Beta3 integrin cell surface interactions:  0 common transcripts | *FN1* | *FBN1* |
|  | *COL1A1* | *THBS1* |
|  |  | *SDC4* |
